# Bacterial Composition of the Human Upper Gastrointestinal Tract Microbiome Is Dynamic and Associated with Genomic Instability in a Barrett’s Esophagus Cohort

**DOI:** 10.1371/journal.pone.0129055

**Published:** 2015-06-15

**Authors:** Alevtina Gall, Jutta Fero, Connor McCoy, Brian C. Claywell, Carissa A. Sanchez, Patricia L. Blount, Xiaohong Li, Thomas L. Vaughan, Frederick A. Matsen, Brian J. Reid, Nina R. Salama

**Affiliations:** 1 Molecular and Cellular Biology Graduate Program, University of Washington, Seattle, Washington, United States of America; 2 Human Biology, Fred Hutchinson Cancer Research Center, Seattle, Washington, United States of America; 3 Divisions of Public Health Sciences, Fred Hutchinson Cancer Research Center, Seattle, Washington, United States of America; 4 Department of Epidemiology, University of Washington, Seattle, Washington, United States of America; 5 Department of Microbiology, University of Washington School of Medicine, Seattle, Washington, United States of America; 6 Department of Medicine, University of Washington School of Medicine, Seattle, Washington, United States of America; 7 Program in Computational Biology, Fred Hutchinson Cancer Research Center, Seattle, Washington, United States of America; 8 Department of Genome Sciences, University of Washington, Seattle, Washington, United States of America; Shiga University of Medical science, JAPAN

## Abstract

**Background:**

The incidence of esophageal adenocarcinoma (EAC) has increased nearly five-fold over the last four decades in the United States. Barrett’s esophagus, the replacement of the normal squamous epithelial lining with a mucus-secreting columnar epithelium, is the only known precursor to EAC. Like other parts of the gastrointestinal (GI) tract, the esophagus hosts a variety of bacteria and comparisons among published studies suggest bacterial communities in the stomach and esophagus differ. Chronic infection with *Helicobacter pylori* in the stomach has been inversely associated with development of EAC, but the mechanisms underlying this association remain unclear.

**Methodology:**

The bacterial composition in the upper GI tract was characterized in a subset of participants (n=12) of the Seattle Barrett’s Esophagus Research cohort using broad-range 16S PCR and pyrosequencing of biopsy and brush samples collected from squamous esophagus, Barrett’s esophagus, stomach corpus and stomach antrum. Three of the individuals were sampled at two separate time points. Prevalence of *H*. *pylori* infection and subsequent development of aneuploidy (n=339) and EAC (n=433) was examined in a larger subset of this cohort.

**Results/Significance:**

Within individuals, bacterial communities of the stomach and esophagus showed overlapping community membership. Despite closer proximity, the stomach antrum and corpus communities were less similar than the antrum and esophageal samples. Re-sampling of study participants revealed similar upper GI community membership in two of three cases. In this Barrett’s esophagus cohort, *Streptococcus* and *Prevotella* species dominate the upper GI and the ratio of these two species is associated with waist-to-hip ratio and hiatal hernia length, two known EAC risk factors in Barrett’s esophagus. *H*. *pylori*-positive individuals had a significantly decreased incidence of aneuploidy and a non-significant trend toward lower incidence of EAC.

## Introduction

The incidence of esophageal adenocarcinoma (EAC) has increased by 463% from 1979 to 2004, making it one of the most rapidly increasing cancers in the United States [1]. Barrett’s esophagus (BE) is the only known precursor of EAC and is characterized by replacement of the stratified squamous epithelium with a metaplastic columnar epithelium. Barrett’s epithelium secretes bicarbonate and mucus, which may be a protective adaptation against gastroesophageal reflux disease. Continued acid exposure and subsequent inflammation has been suggested to induce proliferation of BE cells, which may contribute to the development of EAC [2]. *Helicobacter pylori*, a Gram-negative bacterium, has a well-documented role in the development of gastric and duodenal ulcers, as well as gastric adenocarcinoma [3]. Intriguingly, several studies have shown that infection with *H*. *pylori*, is inversely correlated with the development of EAC [4–7]. While the prevalence of *H*. *pylori* carriage worldwide remains at about 50%, infection rates and incidence of gastric adenocarcinoma have declined sharply in the United States and Europe [8]. This decline preceded the increase in EAC incidence. Inflammation caused by chronic *H*. *pylori* infection can lead to gastric atrophy and loss of acid-producing parietal cells, which predisposes the stomach to cancer [9]. Some studies have suggested that the subsequent hypochlorhydria results in a refluxate that is less damaging to the esophageal epithelium and thus less likely to induce progression to EAC [10]. A more recent case-control study involving over 600 participants found that *H*. *pylori* mediated protection is independent of gastric atrophy, suggesting other consequences of *H*. *pylori* infection should be considered [5]. In the majority of *H*. *pylori* positive individuals, *H*. *pylori* was the most abundant species detected in stomach biopsies [11,12], but the ability of this organism to interact with tissues in the esophagus and the role(s) of other bacteria present in the stomach and esophagus in modifying disease risk at these sights remain unclear.

While the microbiome of the oral cavity and the lower GI tract have been more thoroughly investigated, relatively little is known about the microbial communities that reside within the esophagus and the stomach. A recent study surveying bacteria present in the oral cavity, lower GI and skin in healthy human volunteers showed that the mouth is dominated by members of the *Firmicutes* and *Proteobacteria* phyla, with *Bacteroidetes* being the third most common. The gut microbiome, on the other hand, was primarily dominated by *Bacteroidetes* and to a lesser extent, *Firmicutes* [13]. Another study comparing throat, stomach and fecal microbiota in healthy individuals concluded that stomach and throat communities are more closely related (in the absence of *H*. *pylori* infection) than lower GI communities as measured by UniFrac distance, a phylogeny based metric. Although the *Firmicutes* and *Bacteroidetes* phyla are well represented in the upper and lower gastrointestinal tract, their members differ. The prominent genera in the upper GI are *Streptococcus*, *Gemella* and *Prevotella*, while the lower GI is dominated by *Ruminococcus*, *Clostridium*, *Eubacterium* and *Bacteroides* [11].

The microbial residents of the stomach and esophagus differ from those found in the mouth and the lower GI tract. Using a 16S rRNA gene clone library approach, Bik and colleagues isolated 128 different phylotypes from corpus and antrum biopsies. Most of the sequences were assigned to *Proteobacteria*, *Firmicutes*, *Actinobacteria*, *Bacteroidetes* and *Fusobacteria* phyla. A subset of the patients sampled in the study were confirmed *H*. *pylori* carriers and in these individuals *H*. *pylori* was the most abundant species detected [12]. One of the first studies to use a culture independent method to survey the microbiome of the distal esophagus in healthy individuals determined that the most prevalent genera residing at this site are *Streptococcus* (39%), *Prevotella* (17%) and *Veillonella* (14%) [14]. Another recent study concluded that individuals with Barrett’s esophagus or esophagitis are more likely to harbor Gram-negative anaerobic or microaerophilic organisms, such as *Prevotella*, *Veillonella*, *Haemophilus*, *Neisseria* and *Rothia*. Healthy individuals, on the other hand, were much more likely to be dominated by *Streptococci* species [15].

In the present study, we sought to investigate the relationships between microbial communities found in the distinct tissues of the esophagus and stomach in a Barrett’s esophagus cohort undergoing regular endoscopic surveillance. We compared tissue sampling methods to determine which would maximize recovery of bacterial DNA from upper GI samples. Mucosal brush sampling allowed us to enrich for species abundance and diversity. We reliably mapped greater than 90% of the pyrosequencing reads of amplified 16S rRNA gene fragments down to the species or genus level and found that *Streptococcus* and *Prevotella* species were the most abundant organisms detected in the upper GI tract. We also observed an association between the *Streptococcus* to *Prevotella* species ratio and known risk factors for development of Barrett’s esophagus. We specifically examined the association of *H*. *pylori* with EAC disease progression. Almost all participants in our study utilize one or more acid suppression medications, allowing us to begin addressing *H*. *pylori*’s protective role independent of its role in acid suppression. We found that *H*. *pylori* positive individuals had a decreased incidence of aneuploidy in Barrett’s esophagus tissue.

## Results

### Mucosal brush samples enhance detection of bacterial diversity in the esophagus and stomach

**Fig 1:**
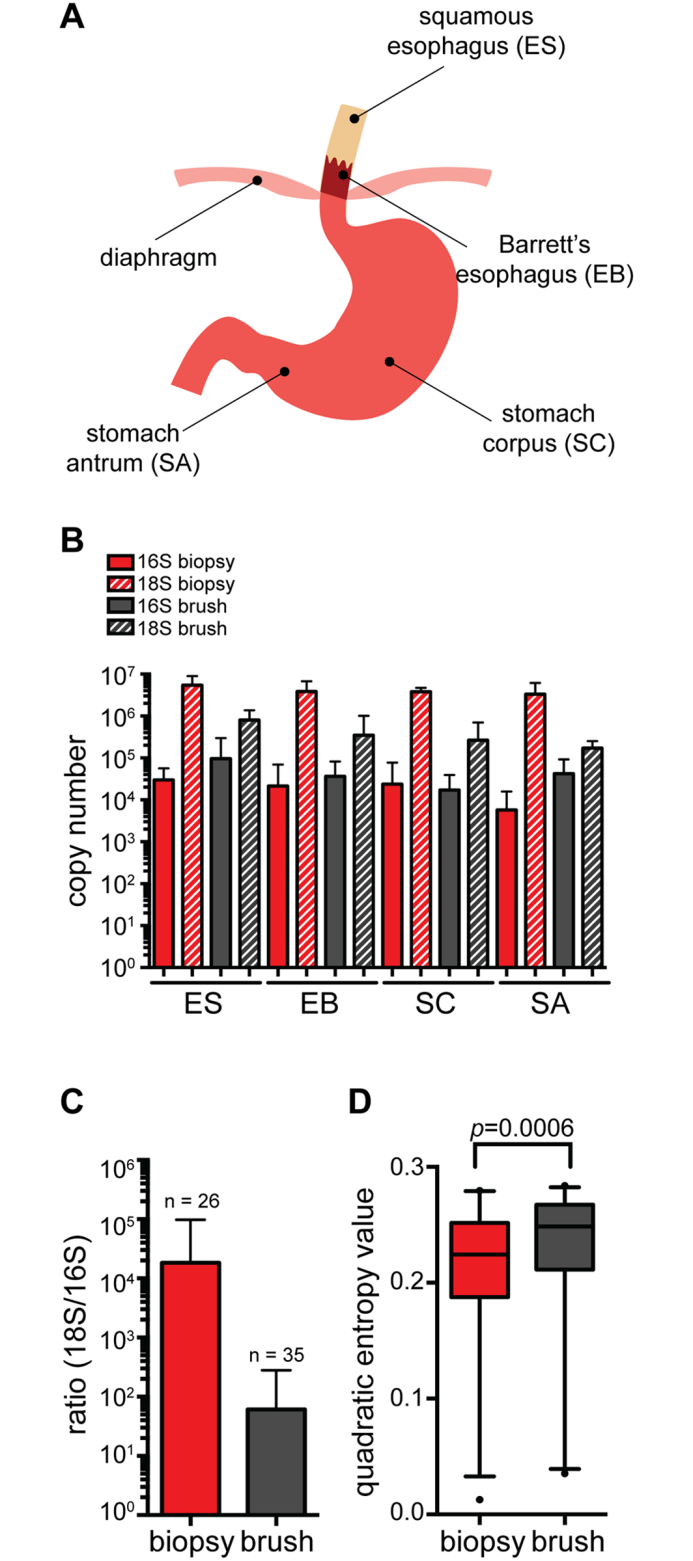
Brush sampling of the upper gastrointestinal tract enriches for bacterial abundance and diversity. (A) Diagram of the human upper gastrointestinal tract indicating regions sampled via biopsy or brush collection method. Anatomic sites were abbreviated with the first and second letter indicating the sampled organ and intra-organ tissue, respectively (ES—squamous esophagus; EB—Barrett’s esophagus; SC—stomach corpus; SA—stomach antrum). (B) Total bacterial versus human DNA recovered from biopsy or brush samples segregated by anatomical site as measured by qPCR and plotted as copy number of bacterial 16S rRNA gene and human 18S rRNA gene. Error bars indicate standard deviation from the mean. (C) Ratio of human 18S rRNA to bacterial 16S rRNA copy number in all biopsy (n = 26) or brush (n = 35) samples. Error bars indicate standard deviation from the mean. (D) Species diversity in biopsy and brush samples as measured by quadratic entropy analysis. The central line within each box represents the median of the data, the whiskers represent the 5^th^ and 95^th^ percentiles and data outside that range are plotted as individual points. Statistical difference between biopsy and brush samples was measured by Wilcoxon rank sum test with continuity correction (p = 0.000594).

To explore bacterial community composition in the upper GI tract in the context of Barrett’s esophagus, we took advantage of the longstanding Seattle Barrett’s Esophagus Research Program (SBERP). SBERP has undertaken surveillance biopsy sampling of participants with Barrett’s esophagus to study the evolution of esophageal adenocarcinoma over several decades. We collected upper GI samples from a subset (n = 12) of SBERP participants (Table 1). To determine the optimal method for surveying the upper GI, we used two sampling methods: tissue biopsies and mucosal brush samples using cytology brushes. One possible advantage of brush sampling relates to cross contamination between samples. The brush resides in a protective sheath while it is threaded through the endoscope channel, is deployed at the site of sampling and is then re-sheathed before being retracted through the endoscope. We also hypothesized that brush sampling would enrich for bacterial cells that, presumably, are exclusively associated with the epithelial surface. The order of sample collection was Barrett’s esophagus, normal squamous esophagus, stomach antrum and stomach corpus (Fig 1A). In cases where both biopsy and brushes were collected, the biopsy samples were collected first and then a brush sample was collected at the same endoscope position. Biopsy forceps were rinsed in sterile water between samples.

**Table 1:** Participant Demographics.

Participant ID	Diagnosis	Sex	Age (yrs)	Acid suppression (number)	Antibiotic	Steroid	NSAID	Statin
**P1**	LG^a^ dysplasia	M	55	Y (1)	N	N	Y (1)	N
**P2**	Metaplasia	M	67	Y (1)	N	N	Y (1)	Y (1)
**P2** ^**b**^	Metaplasia	M	69	N	N	N	Y (1)	Y (1)
**P3**	Metaplasia	F	64	N	N	N	N	N
**P4**	Metaplasia	M	67	Y (1)	N	N	Y (1)	Y (1)
**P5**	GERD	M	71	Y (1)	N	Y (1)	Y (1)	Y (1)
**P6** ^**c**^	Metaplasia	F	80	Y (1)	N	N	Y (1)	Y (1)
**P7**	HG^a^ dysplasia	M	52	Y (2)	N	N	N	N
**P7^b^**	LG dysplasia	M	52	Y (2)	N	N	N	N
**P8**	GERD	M	75	Y (1)	N	N	Y (1)	Y (1)
**P9**	HG dysplasia	M	72	Y (1)	N	N	Y (1)	Y (1)
**P9** ^**b**^	LG dysplasia	M	75	Y (1)	N	N	Y (2)	N
**P10**	LG dysplasia	M	62	Y (1)	N	N	Y (1)	N
**P11**	LG dysplasia	M	62	Y (1)	Y (1)	N	N	N
**P12**	Metaplasia	M	65	Y (1)	N	N	Y (1)	N

^a^ LG = low grade HG = high grade

^b^ Denotes samples collected at a second time point (P2 [t = 2 years]; P7 [t = 4 months]; P9 [t = 3 years])

^c^ Denotes *H*. *pylori*-positive participant

To identify bacteria present in each sample, we isolated total DNA and used broad range PCR amplification of the 16S rRNA with subsequent 454 pyrosequencing of the amplified products. Operational taxonomic units (OTUs) were defined by phylogenetic classifications (not percent identity), where ambiguous assignment to a set of taxonomic groups was considered to be a classification. We compared DNA recovered from tissue biopsy versus cytology brushes. Consistent with previous findings in the stomach and esophagus [12,14,15], tissue biopsies yielded bacterial DNA representing 282 OTUs, but contained a very high ratio of human to bacterial DNA (as high as 100000:1). Compared to biopsies, brush sampling yielded up to one log increase in bacterial 16S copy per sample and lowered human DNA (measured by 18S) recovery by up to two logs (Fig 1B and 1C). For participants where we obtained simultaneous brush and biopsy samples, brushes consistently yielded all of the OTUs found in the biopsy samples, as well as additional OTUs (S1 Table and S1 Fig). The dataset generated from our study contained a total of 296,042 reads with 67.8% of sequences classified at a species level, 23.0% at a genus level and 9.2% at a higher taxonomic level. We did not observe a statistically significant difference between the number of reads detected at each of the sampled sites (S2 Fig) or the number of OTUs in technical replicate samples (S2 Table). Furthermore, the proportion of different OTUs was consistent between the two sampling methods (S2 Fig) and brush and biopsy samples from the same patient showed co-clustering by KR distance metric (S2 Fig). However as shown in Fig 1D, diversity measured by brush sampling was greater than with biopsy sampling, likely because more OTU were detected. Our data suggest that brush sampling is superior to biopsies for maximizing the quantity and quality of recovered bacterial DNA. Thus, in subsequent analyses, we utilized brush samples when available.

### Bacterial communities in the upper GI tract display high inter-individual variation but overlapping community membership between sites

**Fig 2:**
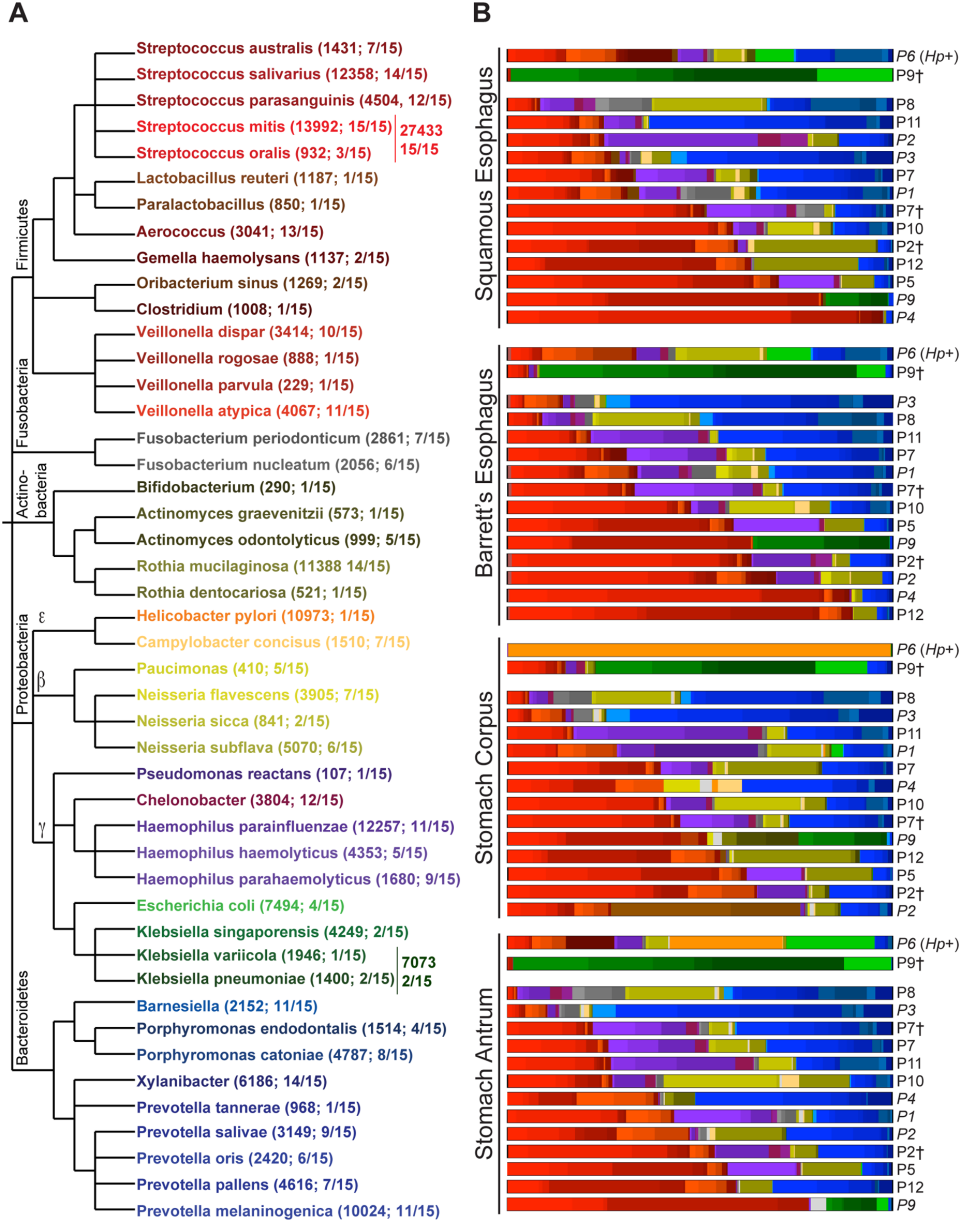
Members of the *Firmicutes* or *Bacteroidetes* phyla dominate the upper gastrointestinal tract microbiome. (A) Phylogenetic relationship of the top 45 OTUs recovered from each of the four sites sampled in individual participants. Respective phyla are noted above main branches of the phylogenetic tree. Numbers in parentheses represent total number of pyrosequencing reads recovered for a given species or genera across all samples followed by the fraction of participants in whom a relative abundance of ≥1.3% of a given species or genera were detected. (B) Species/genera-level profiles of top 45 OTUs detected by 454 sequencing in squamous esophagus, Barrett’s esophagus, stomach corpus and stomach antrum of indicated participants. Data arranged in order of increasing *Firmicutes* dominance. Individual species/genera are color-coded according to scheme presented in (A). Sequencing reads from brush samples were used when available, otherwise, data from biopsy samples are shown. Species reads were normalized to the total number of reads per corresponding site in a given individual. ^†^Denotes samples collected at a second time point (P2 [t = 4 months]; P7 [t = 2 years]; P9 [t = 3 years]); *Hp+* indicates *H*. *pylori*-positive individual. Italicized participant IDs denote data from biopsy samples in cases where brush samples were not available for analysis.

The most abundant bacterial groups observed in our upper GI microbiome samples were members of the *Firmicutes* and *Bacteriodetes* phyla (Fig 2). Two sets of samples deviated from this pattern. One participant had recorded *H*. *pylori* infection based on prior histologic examination. Consistent with previous reports [12], both stomach samples from this participant had abundant *H*. *pylori* reads (99% in stomach corpus). In the stomach antrum sample, in addition to *H*. *pylori* (28% of total reads), a high proportion of other *Proteobacteria* were detected including *Enterobacteriaceae* [exclusively *E*. *coli* and *Shigella*] (Fig 2B). While *H*. *pylori* was also detected in the esophageal samples (5% of total reads), the majority of bacterial reads from the esophageal sites belonged to the *Firmicutes* and *Bacteriodetes* phyla. The second set of outliers constituted samples abundant in *Enterobacteriaceae* from all four sites of individual P9 taken at a 3-year follow-up appointment. With the exception of *Shigella* species, all of the *Enterobacteriaceae* species represented at the 3-year time point were also present at the initial upper GI sampling (t = 0) although at a 10–1000 fold lower abundance (Fig 2B and S3 Table).

**Fig 3:**
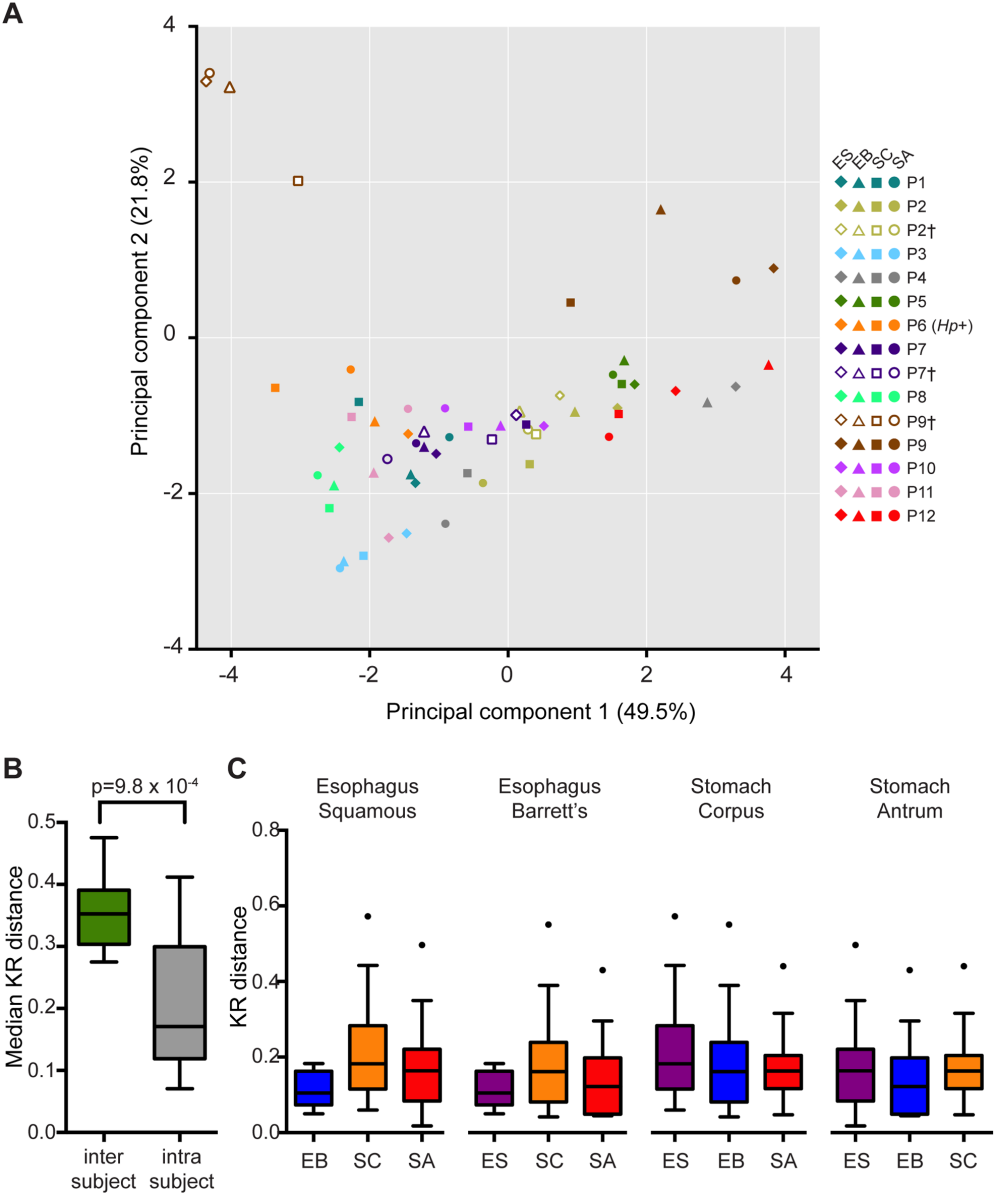
Phylogenetic sample profiles are most similar within individuals rather than across anatomic sites. (A) Principal component analysis of phylogenetic similarity among samples from each of the four anatomic sites of indicated participants. The number in parentheses corresponds to the percent variance of the data assigned to each indicated principal component. (B) Median KR distance across all samples between individuals (inter) and across sites within individuals (intra). Statistical significance was determined by Wilcoxon rank sum test with continuity correction. (C) Intra-individual KR distance of the three anatomic sites relative to the site indicated at the top of each graph. For B & C, the central line within each box represents the median of the data, the whiskers represent the 5^th^ and 95^th^ percentiles and data outside that range are plotted as individual points.

We used principal component analysis to examine the relatedness of our samples. As shown in Fig 3A, the majority of patient samples tended to cluster together. Overall, samples from the same individual were more phylogenetically similar to each other than to samples from the same anatomic site in a different individual (Fig 3B). Among samples within an individual, the stomach corpus samples tended to be the most distinct, while samples from the stomach antrum and esophagus were more similar to each other (Fig 3C). Thus the acidic environment of the corpus may drive the distinct composition at this site but does not prevent seeding of the stomach antrum with organisms present further upstream in the GI tract.

### Community composition similarity with replicate sampling

**Fig 4:**
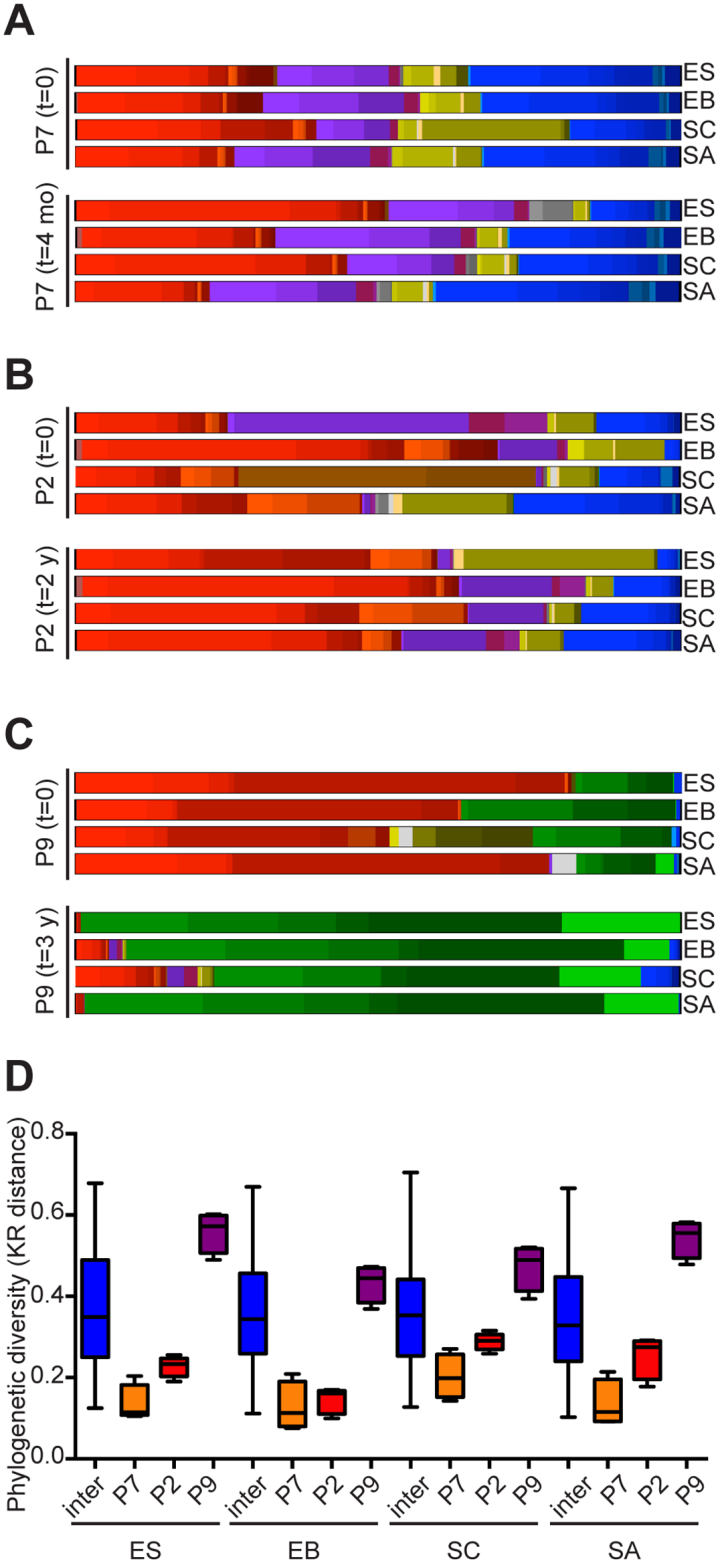
Upper gastrointestinal microbiome similarity with replicate sampling. (A–C) Species/genera-level profiles of microbiota detected by 454 sequencing in squamous esophagus, Barrett’s esophagus, stomach corpus and stomach antrum of individuals P7 at the time of first [t = 0] and second sample collection [t = 4 months] (A), P2 at t = 0 and t = 2 years (B) and P9 at t = 0 and t = 3 years (C). Individual species/genera are presented according to coloring scheme described in Fig 2 (D) Phylogenetic KR distance between (inter) samples from participants P2, P7 and P9 at both time points and within those individuals comparing the 1^st^ and 2^nd^ time points from the indicated anatomic site. The central line within each box represents the median and the whiskers represent the minimum and maximum values.

Three participants in our study were sampled at two time points. The upper GI of individuals P7, P2 and P9 were sampled 4 months, 2 and 3 years apart, respectively. Although the total number of reads per sample varied between individuals across time, particularly when biopsy samples from the initial collection were compared to brush sampling at the second time point, the number of represented species remained stable over time (S3 Table). The upper GI microbiome of individual P7 showed the least amount of change over a 4 month time period (Fig 4A and 4D). Individual P2 (surveyed 2 years apart) showed greater fluctuation in the relative abundance of certain species than P7, but did not display a dramatic shift in microbial community members (Fig 4B and 4D). Individual P9, on the other hand, showed a dramatic expansion in the members of the *Enterobacteriaceae* phylum (Fig 4C). Moreover, a second set of samples obtained at each site from individual P9 were more phylogenetically distinct with respect to the first set than the average inter-subject difference for all samples where two time point collections were available (Fig 4D).

To further examine changes in bacterial community composition among these samples, we narrowed our analysis to the OTUs that increased or decreased in frequency by ≥ 5% between the two time points sampled. We chose 5% as the threshold because the average change in OTU relative abundance between the initial and subsequent sampling was 1.1% with a standard deviation of 3.9%. This allowed us to focus on the OTUs that exhibited a change that was 1 standard deviation or more away from the mean (S3 Table). Of the sixty-one OTUs that changed by ±5% across all sites, fifty were found in either individual P9 or P2. Interestingly, in individual P9 *Shigella* spp. were undetectable at the initial sampling but were detected at all four sites at the second time point with the most dramatic increase (9.6%) seen in the squamous esophagus tissue sample. Similarly, in individual P2 *Neisseria flavescens/subflava* were not detected initially, but were subsequently detected at all four sites with the greatest difference in relative abundance (5.5%) observed in Barrett’s esophagus tissue. Individual P2 also exhibited a loss of *Lactobacillus* and *Paralactobacillus spp*. across all four sites, but most dramatically in the stomach corpus (*Lactobacillus reuteri* and *Paralactobacillus* decrease in relative abundance by 23.8% and 13.9%, respectively). We speculate that this change may have occurred because individual P2 discontinued acid suppression medication (Omeprazole) prior to collection of the second time point specimens. It is unclear, however, how long this participant abstained from proton-pump inhibitor treatment. Indeed, a recent study comparing the composition of the upper GI microbiome before and after PPIs found an increase in members of the *Lactobacillales* family after PPI treatment was initiated [16].

Although the clinical status of individual P7 did not change over a 4-month time period (metaplasia at the initial and follow-up sampling), individuals P2 and P9 both regressed from a diagnosis of high grade to low grade dysplasia. This change in clinical diagnosis was accompanied by an expansion in members of the *Proteobacteria* phylum. Individual P2 experienced an expansion of *Betaproteobacteria* (*Neisseria* species) and *Gammaproteobacteria* (*Chelonobacter* and *Haemophilus* species). In individual P9, however, *Klebsiella* and *Escherichia* species, members of the *Gammaproteobacteria* class, underwent an expansion (S3 Table).

### *Streptococcus* to *Prevotella* ratio is associated with waist to hip ratio and hiatal hernia length, two known Barrett’s esophagus and EAC risk factors

**Fig 5:**
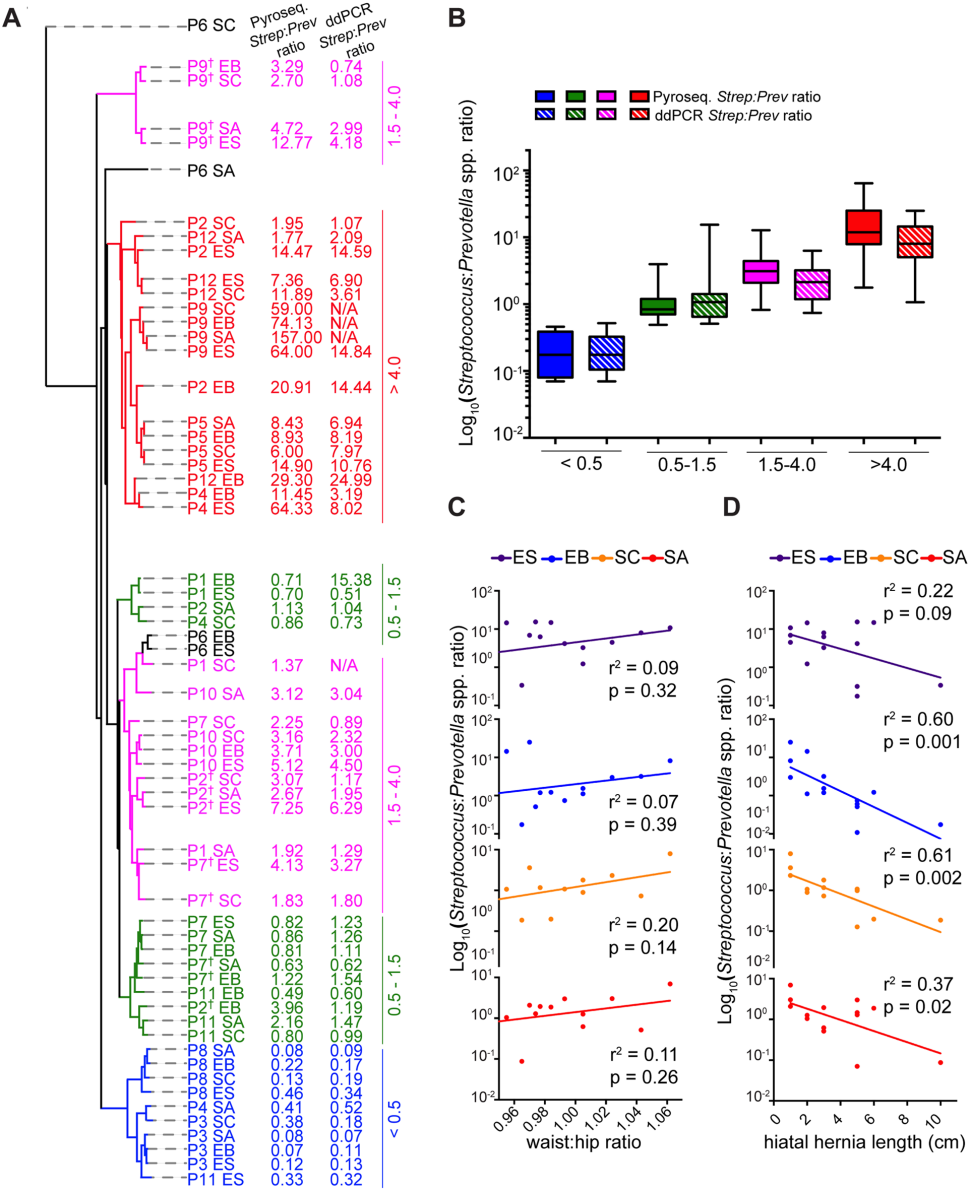
*Streptococcus* to *Prevotella* species ratio corresponds to phylogenetic distance sample clustering and correlates with Barrett’s esophagus risk factors. (A) Cluster analysis of KR distances between microbial communities of individual study samples. Pyroseq. *Strep*:*Prev* ratio was calculated using relative abundance of mapped reads for all *Streptococcus* and *Prevotella* species as determined by pyrosequencing. ddPCR *Strep*:*Prev* ratio was calculated using copies/μl of a *Streptococcus* or *Prevotella*-specific 16s rRNA gene segment as determined by droplet digital PCR. Samples color-coded based on the majority of calculated Pyroseq. *Strep*:*Prev* ratios in a group being <0.5 (blue), 0.5–1.5 (green), 1.5–4.0 (magenta) or >4.0 (red). (B) Boxplots comparing *Streptococcus* to *Prevotella* ratio as determined by pyrosequencing and ddPCR. The central line within each box represents the median of the data, the whiskers represent the 5^th^ and 95^th^ percentiles and data outside that range are plotted as individual points. (C) Relationship of *Streptococcus* to *Prevotella* ratio (measured by ddPCR) and waist-hip ratio of all male participants segregated by anatomic site. Strength of association between these two variables was determined by Pearson’s correlation test with correlation coefficient squared (r^2^) values as indicated. (D) Relationship of *Streptococcus* to *Prevotella* ratios (measured by ddPCR) and hiatal hernia length in all participants segregated by anatomic site. Strength of association tween these two variables was determined by Pearson’s correlation test with correlation coefficient squared (r^2^) values as indicated.

Given that *Firmicutes* or *Bacteroidetes* constituents dominated the majority of the samples in our study, we wanted to investigate the key species in our samples within each of these phyla. Based on the relative abundance of all members of the *Firmicutes* and *Bacteriodetes* phyla, we determined that either the *Streptococcus* (*Firmicutes*) or the *Prevotella* (*Bacteriodetes*) genera dominate within individuals and across anatomic sites (Fig 2). We preformed a cluster analysis based on the phylogenetic placement of sequences for all study samples and observed that participant samples roughly segregated into four groups based on their *Streptococcus* to *Prevotella* ratios [ratio <0.5, 0.5–1.5, 1.5–4.0 & >4.0] (Fig 5A). We confirmed the *Streptococcus*:*Prevotella* ratios determined through pyrosequencing by quantifying all *Streptococcus* and *Prevotella* species in our samples using droplet digital PCR. In order to do so, we designed pan-*Streptococcus* and pan-*Prevotella* primers that bind to a species-conserved region of the 16S rRNA outside of the sequence used to amplify the 16S rRNA for our pyrosequencing studies (S3 Fig). There was no statistical difference between the *Streptococcus*:*Prevotella* ratios as determined by pyrosequencing and ddPCR (Fig 5B). We then investigated correlations between the *Streptococcus*:*Prevotella* ratios and clinical features of the participants in our study. A high waist-to-hip ratio has been associated with an increased risk for progression to EAC in our study cohort [17] as well as others [18]. The presence of a hiatal hernia is independently associated with an increased risk of Barrett’s esophagus [19]. We found that *Streptococcus*:*Prevotella* ratios in the stomach corpus positively correlated with waist-to-hip ratios of the male study participants (Fig 5C), although the correlation was not statistically significant. We also found a highly statistically significant inverse correlation of *Streptococcus*:*Prevotella* ratios in the stomach corpus (p = 0.002) and Barrett’s esophagus (p = 0.001) with hiatal hernia length, and a slightly weaker inverse correlation in the stomach antrum (p = 0.02) (Fig 5D). Notably, we did not observe a similar correlation between waist-to-hip ratio and hiatal hernia length in this population ([r^2^ = 0.12] S4 Fig), indicating that these two clinical parameters are independently associated with *Streptococcus*:*Prevotella* ratios. An increased Barrett’s segment length has been associated with progression to high grade dysplasia and EAC [20]. We noted a negative correlation between the *Streptococcus*:*Prevotella* ratio in the stomach corpus and increased Barrett’s segment length ([r^2^ = 0.28, p = 0.07] S4 Fig). We observed no appreciable correlations between *Streptococcus*:*Prevotella* ratio and clinical diagnosis, inflammation score, age or tobacco use (S4 Fig).

### *H*. *pylori* carriage is associated with decreased genomic instability among individuals with Barrett’s esophagus

**Fig 6:**
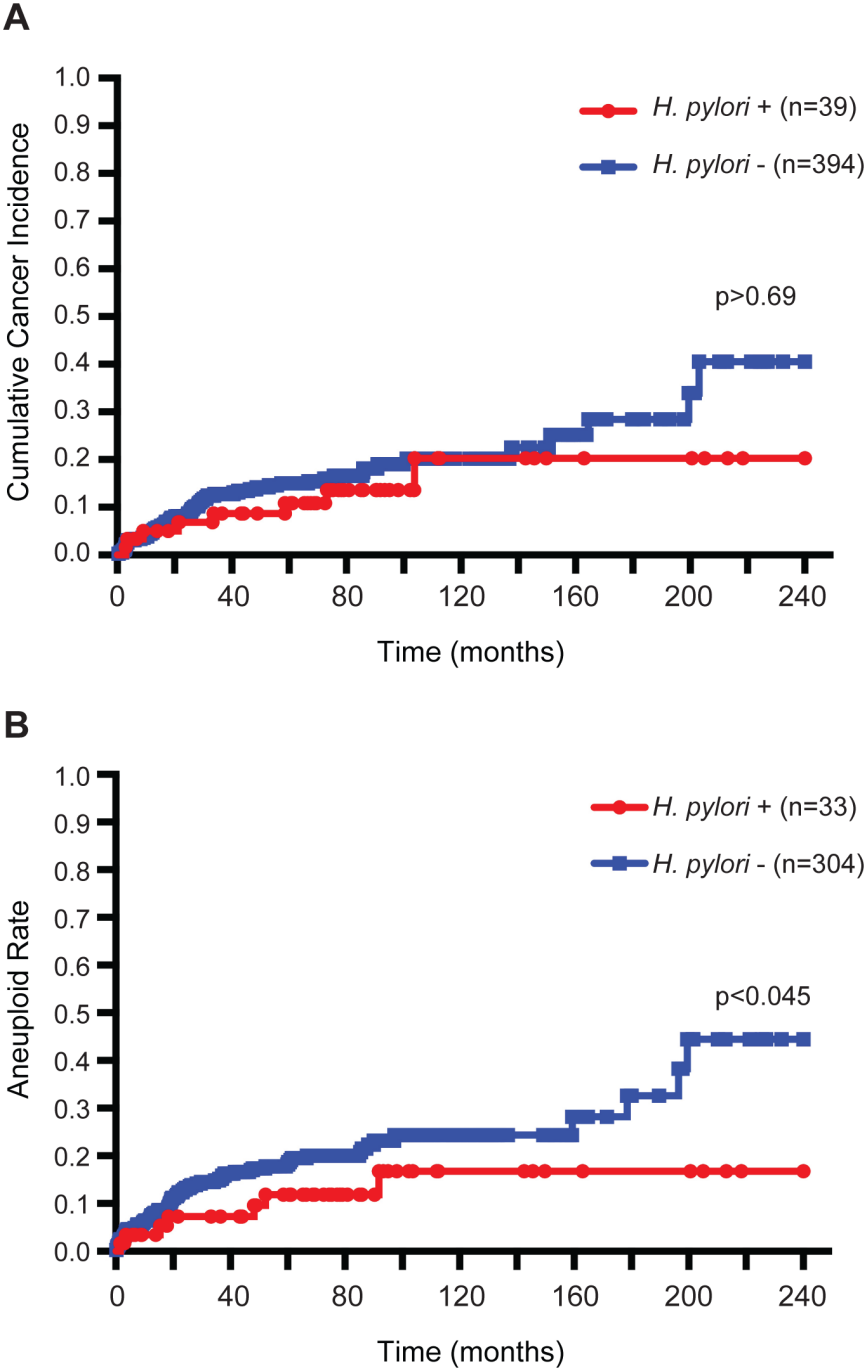
Incidence of cancer and aneuploidy in Seattle Barrett’s Esophagus Research Program cohort. Kaplan-Meier curves for participants within the cohort for (A) cancer incidence (n = 433, 39 infected) and (B) aneuploidy as measured by DNA content flow cytometry (n = 337, 33 infected). *H*. *pylori* infection was assessed by histology of antral biopsies. Note aneuploidy information was not available for all research participants. Statistical significance was determined using the Log-rank test.

Limited by sample size, we were unable to explore the effect of *H*. *pylori* carriage on bacterial members of the esophagus. However, given that we detected *H*. *pylori* reads in esophageal samples of individual P6, we wondered if *H*. *pylori* status correlated with disease progression in the larger Barrett’s esophagus cohort. As of 2004, baseline *H*. *pylori* infection status was available from histologic evaluation of stomach antrum biopsies for 433 participants. Prevalence of *H*. *pylori* infection is low in this cohort (9%). As shown in Fig 6A, a trend towards a lower incidence of esophageal adenocarcinoma development among *H*. *pylori* positive participants was observed (Log-rank test p>0.69), but was not statistically significant. Genomic instability has emerged as an important predictor of subsequent cancer development in the context of Barrett’s esophagus [21]. Average DNA content measured by flow cytometry was available for a subset (78%) of participants for which *H*. *pylori* infection status had been measured. Interestingly, *H*. *pylori* positive participants showed a lower incidence rate of DNA content aneuploidy than *H*. *pylori* negative participants, which was statistically significant (Fig 6B; Log-rank test p<0.045). These results suggest that *H*. *pylori* may directly or indirectly influence cancer progression in the esophagus.

## Discussion

Here, we found that infection with *Helicobacter pylori* in a Barrett’s esophagus cohort was associated with decreased incidence of aneuploidy, a measure of genomic instability that predicts progression to esophageal adenocarcinoma [21]. Infection with *H*. *pylori*, and particularly *cagA*-positive strains, is associated with development of gastric adenocarcinoma [22], but is inversely associated with development of EAC [5–7]. CagA protein is delivered to the host epithelial cell via the *cag*-type IV secretion system, where it is phosphorylated and activates a variety of oncogenic pathways including the host oncoprotein SHP-2 [23]. Consistent with previous studies [12], we found that, when present, *H*. *pylori* was 99% and 24% abundant in the stomach corpus and antrum, respectively. Intriguingly, we also detected *H*. *pylori* reads in both of the sampled esophageal sites (5% relative abundance). This is consistent with a recent study utilizing a culture-based method to probe esophageal microbiota where *H*. *pylori* was detected in biopsies collected from healthy, reflux esophagitis and Barrett’s esophagus tissues [24]. These findings raise the possibility that *H*. *pylori* may be directly interacting with host epithelial cells in the esophagus and/or other bacterial members residing in the tissue. Whether *H*. *pylori* is able to deliver CagA protein to esophageal epithelial cells by the same mechanism as in the stomach remains unclear. Mutations in SHP2 and aberrant activation of this protein have been implicated in squamous head and neck cancer [25], but not esophageal adenocarcinoma. Due to the limited number of *H*. *pylori* positive samples in our study we were unable to evaluate CagA status in our study cohort. However, whether CagA plays a role in EAC remains an important and unanswered question. Perhaps CagA protein is not translocated or phosphorylated in the context of the esophagus and *H*. *pylori* interaction with the host cells is tissue specific. Further work is necessary to address these questions and shed more light on *H*. *pylori*’s role in development of EAC.

Numerous studies in recent years have contributed to our understanding of the human microbiome, however, the upper GI has not received as much research focus as sites which are easier and less invasive to sample (e.g. skin, oral mucosa, lower GI via stool sampling, etc.). Questions remain whether the esophagus and the stomach are host to distinct microbial communities or if these sites are an extension of each other. It is also unclear whether the upper GI communities are more similar to bacteria found in the oral cavity or the lower GI tract. The overlap in membership of the oral, pharyngeal, esophageal and intestinal microbiota has been well documented and it has been suggested that the oral mircobiota substantially contributes to the seeding of downstream sites in the gastrointestinal tract [11,12,26–30]. The oral cavity is dominated by *Streptococcus* and *Veillonella* (*Firmicutes*), *Neisseria* and *Haemophilus* (*Proteobacteria*) and *Prevotella* (*Bacteroidetes*) species, which vary less over time than gut communities. The gut microbiome is more diverse and consists largely of *Bacteroides* and *Prevotella* (*Bacteroidetes*), as well as *Ruminococcus* (*Firmicutes*) species [13,26,31,32]. Our findings suggest that esophageal and stomach communities are more similar in composition to the oral cavity than the lower GI and support the idea that the upper GI is seeded, in part, by oral communities. One study investigating the composition of bacterial communities in the oral cavity, gut, skin, nostril, hair on head and external auditory canal observed that the strongest sample clustering occurred by anatomic site rather than sex, individual or time point [13]. Moreover, distinct distribution of species at different oral sites (i.e. keratinized vs. non-keratinized squamous epithelium and gingival plaque) have been suggested to be shaped by localized environmental factors [26]. We, however, did not observe a substantial difference in microbial community membership or diversity in the squamous esophagus versus bacteria associated with columnar Barrett’s epithelium. Among the twelve individuals studied, sequences from the various upper GI sites sampled within a given individual were phylogenetically closer to each other than they were to sequences from the same site in another individual. Of the upper GI sites sampled, the stomach corpus tended to be most distinct and antrum communities were more similar to those found in the esophagus, rather than the stomach corpus that is immediately proximal. While association with different types of epithelial surfaces did not appear to select for different community membership, the low pH associated with the stomach corpus compared to the antrum and esophagus may be a major factor underlying intra-individual variation in our study. Indeed, changes in pH have been shown to modify microbial communities in the stomach and esophagus of a Barrett’s esophagus and esophagitis cohort [16].

Temporal studies of the gastrointestinal microbiota have shown that commonly shared taxa across individuals at different body sites, referred to as the core microbiome, remain stable over time [13,31,33–35]. In the lower GI tract, factors such as long-term diet and weight stability are strong predictors for maintaining the taxonomic core [31,34]. Two of the three participants in our study who were sampled at a second time (4 months and 2 years) showed high phylogenetic similarity between samples from the two time points. Interestingly, P2 had stopped PPI therapy at the second time point, yet the community looked quite similar to the first time point with the exception of a few specific species noted above.

A third participant (P9) who was sampled after three years had a microbiome composition that was phylogenetically distinct between the two sampling times. At the initial sampling, the upper GI of individual P9 was dominated by *Streptococci* species with members of the *Klebsiella* and *Escherichia* species present at a much lower abundance. At the second sampling, we observed a substantial expansion in the relative abundance and diversity of the *Enterobacteriaceae* family. A similar expansion of *Enterobacteriaceae* members has not been documented in Barrett’s esophagus or stomach microbiome studies. Given the available data, it is impossible to determine what factors contributed to the microbial shift observed in individual P9. Interestingly, a study investigating the role of fecal microbiota composition and frailty found that a decrease in *Lactobacilli* (*Firmicutes*), *Bacteroides* and *Prevotella* (*Bacteroidetes*) species, accompanied by an increase in *Enterobacteriaceae* species was significantly associated with a higher frailty score in elderly individuals [36,37]. An overall decrease in species richness in the lower GI has also been associated with aging [38].

A handful of recent studies have sought to determine the core microbiome of the upper GI tract in healthy and disease states. One study investigating the microbiome of the esophagus found differences in the microbial composition of healthy individuals versus those with esophagitis or Barrett’s esophagus. They proposed a distinction between a type 1 microbiome of healthy individuals, dominated by *Streptococci* species, and a type 2 microbiome of individuals with esophagitis and Barrett’s esophagus with an increased relative abundance of Gram-negative, anaerobic/microaerophilic species (including *Prevotella*, *Veillonella*, *Haemophilus*, *Neisseria* and *Rothia*) [15]. Another study observed a similar enrichment of *Veillonella*, *Prevotella*, *Fusobacterium* and *Neisseria* in Barrett’s but not normal esophagus [24]. Our data suggest that the relative abundance of *Streptococci* species can vary substantially in individuals with Barrett’s esophagus and that the balance of *Streptococcus* and *Prevotella* species in the upper GI may be associated with Barrett’s esophagus risk factors, such as central obesity [17]. An increase in *Bacteroidetes* species in the lower GI has been linked to an increase in lean body mass. While an increase in *Firmicutes* species, and corresponding decrease in *Bacteroidetes* species, has been linked to obesity [27,39–41]. It has been postulated that members of the *Firmicutes* phylum are better suited for metabolizing short-chain fatty acids and extract more energy from the host diet than their *Bacteroidetes* counterparts [27,42]. It is unclear how well the ratio of *Firmicutes* to *Bacteroidetes* species in the upper GI mirrors that found in the lower GI. However, given our findings, it is possible that the relative abundance of bacterial members in these two phyla may be similarly linked to obesity in the upper GI.

As discussed above, our study was limited by its small sample size. *H*. *pylori* status was known for a larger subset of the Seattle Barrett’s cohort study participants (n = 433) allowing us to evaluate aneuploidy incidence and progression to EAC in relation to *H*. *pylori*, though we only observed a statistically significant association with aneuploidy incidence. We evaluated the rest of the bacterial microbiome of a much smaller subset of the cohort (n = 12) which precluded us from making definitive conclusions regarding the role of other bacterial species present in Barrett’s esophagus and progression to EAC. The small number of individuals evaluated may also have obscured tissue specific associations of bacterial communities that have been observed in other larger studies at other anatomic sites. Our small sample size precludes any conclusions concerning upper GI microbiome stability over time. Future studies will be focused on obtaining more samples from individuals over time, as well as greater representation of shorter (months) and longer (years) timespans between samplings. From a technical standpoint, we were not always able to map 16S reads to a genus or species level. Future studies may benefit from additional metagenomic sequencing platforms that incorporate larger portions of the 16S gene combined with additional genomic sequences and computational analysis methods to improve resolution and distinguish among similar organisms. All of the participants in our study had Barrett’s esophagus, thus we were unable to investigate how the upper GI microbiome differs from that of healthy individuals. Although we detected *H*. *pylori* in the esophageal samples, this study did not evaluate whether the bacteria were directly interacting with the epithelium or if they were transiently present in the esophagus due to regurgitation of the stomach contents.

Despite the limitations, our study presents several strengths. The prospective nature of the Seattle Barrett’s cohort and regular endoscopic surveillance of participants, allowed us to sample multiple sites in the upper GI tract over time. Although constrained by the number of study samples, we were able to focus our study on characterizing the microbial community in the esophagus and the stomach of individuals with Barrett’s esophagus. Piloting the use of cytology brushes to sample the upper gastrointestinal tract, we found that this method enriches for both bacterial abundance and diversity. Although each of the four anatomic sites sampled were distinct from each other within a given individual (especially the stomach corpus), the high inter-individual variability observed in our study suggests that the microbiome of the esophagus and the stomach is more of a continuous site in terms of bacterial diversity and composition. We also found that *Streptococcus* and *Prevotella* species numerically dominate the upper GI in this study cohort, but the ratio of the two varies from person to person and across anatomic sites. Follow-up studies with a larger sample size will evaluate whether the *Streptococcus*:*Prevotella* ratio is of clinical significance for Barrett’s esophagus and progression to EAC. Our analysis of the upper GI samples from an *H*. *pylori*-positive individual suggest that *H*. *pylori* can be found in the esophagus of infected individuals. Future studies will validate these findings, as well as assess the role *H*. *pylori* plays in the upper GI, particularly, the esophagus. *H*. *pylori* may be directly modifying the esophageal microbial community or it may simply be coming along for the ride in the stomach refluxate. Additionally, it may be interacting with esophageal tissues via different mechanisms than the stomach, which may, in part, explain its inverse association with EAC.

## Materials and Methods

### Ethics statement

Written and oral consent was obtained from each individual prior to sample collection. Institutional Review Board approval was obtained for this study through the University of Washington and Fred Hutchinson Cancer Research Center.

### Study participants and sample collection

Study participants described in the present study (n = 12) are a subset of individuals enrolled in the Seattle Barrett’s Esophagus Research Program (SBERP). Established in 1983, SBERP is a prospective cohort of BE patients that undergo periodic endoscopic surveillance via collection of tissue biopsy samples from the Barrett’s segment as previously described [43,44]. Individuals were recruited to participate in SBERP between 1983–2008 and were included in the study if they provided consent, were at least 18 years of age, were medically fit to undergo endoscopy, and had Barrett’s esophagus but not esophageal adenocarcinoma at baseline endoscopy. If a prospective participant did not have high grade dysplasia at baseline, the length of his/her Barrett’s segment had to be 3 cm or longer. If high grade dysplasia was present, then the Barrett’s segment length requirement did not apply. A subset of the cohort presented in this study is comprised of participants who underwent initial endoscopy between 1985 and 2008 and were in active surveillance between 2009–2012, when our study was conducted. As part of this study, additional samples from normal squamous esophagus, stomach corpus and antrum were collected from each study participant for a total of four samples after an initial survey of the upper GI tract (stomach to esophagus). Esophageal samples were collected first (Barrett’s then squamous) before stomach samples (antrum then corpus) to avoid contamination of the esophagus with stomach bacteria. In addition to tissue biopsies, mucosal tissue was also sampled using a flexible 1.5 mm x 140 cm cytology brush (Boston Scientific, Boston, MA) threaded through one of the endoscope working channels. Normal squamous esophagus, Barrett’s esophagus, stomach corpus and stomach antrum were sampled via the brush method in 9 out 12 participants. Biopsy forceps were washed in sterile water between samples and a new cytology brush was used for each sample. Prior to sample collection, each participant was subject to a short interview in which information regarding changes in his/her medications and habits (i.e. tobacco or alcohol use) was recorded. To obtain a waist-to-hip ratio, a trained staff member measured the waist circumference of each participant at the level of the iliac crest and hip circumference at the largest circumference around the buttocks. The diaphragmatic hiatus, distal end of the tubular esophagus and the squamocolumnar junction (SCJ) were measured at endoscopy, as previously described [45]. Briefly, the distal end was defined as the endoscopic lower esophageal sphincter where the tubular esophagus joins the proximal margin of the gastric folds. The SCJ is defined as the location where the squamous-lined esophagus joins the columnar-lined esophagus. The Barrett's segment length was measured as the distance between the distal end and the SCJ. The hiatal hernia is defined as the distance from the diaphragmatic hiatus and the distal end of the tubular esophagus. Barrett’s esophagus biopsies and control tissues were evaluated and scored individually by a pathologist blinded to participant clinical status. Biopsies were classified as squamous, metaplasia, low-grade dysplasia, high-grade dysplasia or esophageal adenocarcinoma according to the highest degree of abnormality found in the biopsy. The extent of inflammation was similarly evaluated and graded as acute or chronic and mild, moderate or severe. Cell aneuploidy was measured by means of DNA content flow cytometry on nuclei isolated from frozen endoscopic biopsy samples and stained with 4’,6-diamidino-2-phenylindole (10 μg/mL) as described previously [44].

### DNA isolation

Each biopsy and brush sample was collected into a cryovial containing minimal essential media plus 10% DMSO, 5% fetal calf serum, 5mM HEPES and frozen at -80°C until further processing. Prior to DNA isolation, samples were thawed and brush samples unsheathed. Cryovials were then vortexed for 1 min each, centrifuged at 12800 rpm for 2 min and supernatant removed. Total genomic DNA was then isolated using UltraClean Microbial DNA Isolation Kit [MoBio, Carlsbad, CA] according to manufacturer’s instructions. Mock digests containing only the media were used to assess contamination from extraction reagents. DNA extracted from biopsy and brush samples was eluted in 100 μl and 25 μl of UltraClean elution buffer, respectively.

### Quantitative PCR

Total bacterial DNA concentrations (16S rRNA gene copies) in each sample were measured using quantitative PCR. TaqMan broad-range 16S rRNA gene primers and probe used were as follows: forward primer (343F) 5’-TACGGRAGGCAGCAG-3’ [46]; reverse primer (806R) 5’ GGACTACCVGGGTATCTAAT-3’; probe 5’FAM-TKACCGCGGCTGCTGGCAC-TAMRA-3’. The master mix (Applied Biosystems, Carlsbad, CA) contained buffer A (1X), magnesium chloride (3 μM), deoxynucleotide triphosphates (1 μM), forward primer (0.8 μM), reverse primer (1 μM), AmpErase uracil-N-glycosylase (0.05 U), probe (200 μM) and AmpliTaq Gold LD polymerase (2.2 U) per reaction. With the exception of water and Taq polymerase, all reagents were additionally filtered using a Microcon centrifugal filter unit (Millipore, Billerica, MA) with sequential centrifugation at 2000 rpm for 10 min, 4000 rpm for 10 min and 8000 rpm for 5 min. Samples were quantified on a 7900HT sequence detection system (Applied Biosystems, Carlsbad, CA) with *E*. *coli* plasmid ranging from 10^1^ to 10^7^ gene copies used to generate a standard curve. The 18S rRNA gene forward primer (5'-CTCTTAGCTGAGTGTCCCGC-3' [20 μM]) and reverse primer (5'-CTTAATCATGGCCTCAGTTCCGA-3' [20 μM]) were added to SYBR Green real-time PCR master mix (Invitrogen, Carlsbad, CA) and total human DNA concentrations were similarly measured using qPCR in each sample. *E*. *coli* transformed with plasmid expressing full-length 18S rRNA gene were used to generate a standard curve (ranging from 10^2^ to 10^9^ gene copies).

## 454 pyrosequencing and analysis

The V3-V4 16S rRNA hypervariable region was amplified in each study sample using broad-range PCR with 347F and 806R primers, as previously described [46,47]. Three GS FLX+ Titanium forward primers (Roche, Basel, Switzerland) were used in combination to enrich for a broad range of bacterial taxa. Forward primers F1, F2 and F3 were used in a 3:1:1 ratio (F1: 5’-CGTATCGCCTCCCTCGCGCCATCAG**GC**ggaggcagcagtrrggaat-3’; F2: 5’-CGTATCGCCTCCCTCGCGCCATCAG**GC**ggtggctgcagtrrgg-3’; F3: 5’-CGTATCGCCTCCCTCGCGCCATCAG**GC**ggtggcagcagtrrgg-3’), where the uppercase sequence corresponds to the 454 Life Sciences FLX adapter, the bold face sequence represents the GC linker and lowercase sequence, the target specific 16S rRNA sequence. A Titanium reverse primer (5’-CTATGCGCCTTCCAGCCCGCTCAGXXXXXX**GC**ggactaccvgggtatctaat-3’), containing an adapter sequence, 6bp barcode (represented by X) and a GC linker sequence, was added to each sample to allow for multiplexing the sequencing reactions. The barcode sequences used in this study are described in previously published work [48]. Amplified 16S rRNA fragments were PCR purified using Agencourt AMPure beads (Beckman Coulter, Pasadena, CA) and quantified using a Quant-iT PicoGreen dsDNA assay kit (Invitrogen). Samples were diluted to 1 x 10^7^ molecules/μl, pooled (16 samples per pool) and sequenced on a 454 FLX or GS Junior instrument (Roche).

The overall quality of sequencing reads had to meet specific criteria in order to be included in subsequent analyses. Sequences were filtered to include only those having no ambiguous base calls (N’s), at least 228 nucleotides in length, a mean quality score of 25, and a match to a known barcode and primer. A single homopolymer error was allowed in the forward primer. 454 sequences were “placed” on a fixed reference tree built from full-length 16S sequences customized for the stomach and esophagus using pplacer [49] in posterior probability mode. Placed sequences were then classified to the most specific rank possible with high confidence using the pplacer “hybrid2” classifier, which refines a naïve Bayes classifier [50] with a classification based on phylogenetic placement location. When a sequence matched multiple taxa of the same rank with probability >0.2, the sequence was assigned to a combination of all taxa. Mapped reads in our study ranged from 70–28,113 reads with a median 2969 reads per sample. Distances between samples were calculated using the phylogenetic Kantorovich-Rubinstein (KR) metric, also known as the “earth-mover” distance. As described in [51], the KR metric in this context is a slight generalization of weighted UniFrac, allowing assignments to internal edges of the tree and for mass to be split according to probabilistic assignment. Clustering was performed using squash clustering and edge principal components [52], which are methods that take advantage of the underlying phylogenetic tree to provide more interpretable clustering and ordination results. Intra-sample diversity was measured using quadratic entropy [53].

The sequencing data presented in this study has been made publically available through the NCBI Sequence Read Archive (BioProject ID: PRJNA270661 available at http://www.ncbi.nlm.nih.gov/bioproject/270661). We used an HMP developed protocol to remove sequences of human origin from our dataset to protect participant privacy [54]. The protocol entitled “Human Sequence Removal” is available at http://hmpdacc.org/tools_protocols/tools_protocols.php.

### Droplet digital PCR

Total *Streptococcus* and *Prevotella* species were quantified from genomic DNA isolated from study biopsy and brush samples using droplet digital PCR (ddPCR). The following primers and probes were designed or modified for this study in order to amplify a species-specific portion of the 16S rRNA gene distinct from the region amplified for pyrosequencing. *Streptococcus spp*.: forward primer (5’-AGATGGACCTGCGTTGT-3’); reverse primer (5’-TGCCTCCCGTAGGAGT-3’) [55]; probe (5’-FAM-CGATACATAGCCGACCTGAGAGG-BHQ-3’). *Prevotella spp*.: forward primer (5’-GATGCGTCTGATTAGCTTG -3’); reverse primer (5’-CCAATATTCCTCACTGCTG-3’); probe (5’-HEX-CGATACATAGCCGACCTGAGAGG-BHQ-3’). Commercially available genomic DNA from *Streptococcus mitis* and *Prevotella melaninogenica* (ATCC, Manasses, VA) were used as controls to optimize the ddPCR assay. Twenty-five microliter reactions containing 2X master mix (Bio-Rad, Hercules, CA), 20X primer/probe mix (900 nM forward and reverse primer, 250 nM probe) and 1 μl of DNA template were prepared in duplicate for each sample. Reaction droplets were generated by mixing 20 μl of each reaction mixture with 70 μl of droplet generation oil in a DG8 cartridge and emulsified in the QX100 Droplet Generator (Bio-Rad, Hercules, CA). Forty microliters of the completed emulsion were slowly transferred to a Twin.tec semi-skirted 96-well PCR plate (Eppendorf, Hamburg, Germany) and heat-sealed with a foil cover. The following thermal cycler program was used to amplify the species-specific 16S rRNA gene fragment: 10 min at 95°C; 40 cycles of 30 sec at 95°C and 1 min at 55°C; 10 min at 98°C. Droplets containing amplified gene fragments were then acquired using the QX100 Droplet Digital PCR system (Bio-Rad). Positive and negative droplets were distinguished based on probe fluorescence amplitude and quantified using QuantaSoft software (Bio-Rad).

### Statistical analysis

Statistical analysis was performed using R version 3.0.3 (http://www.R-project.org/) [56]. Data was graphed using Prism 6 software for Mac OS X (GraphPad, La Jolla, CA). Statistical significance for Kaplan-Meier curves for incidence of cancer and DNA content aneuploidy was assessed using the Log-rank test. Quadratic entropy was used as a measure of species diversity [57] and the difference between biopsy and brush samples was determined by Wilcoxon ranked sum test. Similarly, the phylogenetic distance was approximated using the KR distance metric [51] and distances were compared within and between individual samples using the Wilcoxon ranked sum test. Pearson’s correlation test was used to determine the relationship between *Streptococcus* to *Prevotella* ratios and hiatal hernia length, waist-to-hip ratio, Barrett’s segment length and participant age. Differences in read counts recovered across sites, as well as differences in 18S and 16S copy number were assessed using Freidman rank sum test. For all statistical tests results were considered significant if the p-value for the test was less than or equal to 0.05. In the case of Pearson’s correlation analysis, an uncorrected p-value threshold of 0.05 was used to determine statistical significance.

## Supporting Information

S1 Fig Observed sequencing depth and DNA recovered at each anatomic site. (A) Number of read counts from all study participants per site sampled. Statistical difference between sites was measured by Friedman rank sum test (p = 0.55). (B) Copy number of bacterial 16S rRNA per site sampled, as measured by qPCR. Statistical difference between sites was measured by Friedman rank sum test (p = 0.004). (C) Copy number of human 18S rRNA per site sampled, as measured by qPCR. Statistical difference between sites was measured by Friedman rank sum test (p = 0.001)
(TIF)

S2 Fig Comparison of OTUs detected in brush versus biopsy samples. (A) Phylogenetic relationship of the top 45 OTUs detected in samples where both a brush and biopsy specimen was available. Numbers in column represent the total number of reads detected for a given species or genera in brush and biopsy samples. (B) Combined species/genera-level profiles of top 45 OTUs detected by 454 sequencing at all four sites sampled via upper endoscopy in indicated participants. Data are color-coded according to scheme presented in (A). Species reads were normalized to the total number of reads per corresponding site in a given individual.^†^ Denotes samples collected at a second time point (P2 [t = 4 months]; P7 [t = 2 years]; P9 [t = 3 years]). (C) Cluster analysis of KR distances between microbial communities detected in brush or biopsy samples of indicated individuals.
(TIF)

S3 Fig Droplet Digital PCR assay for detection of *Streptococcus* and *Prevotella* species in study samples. (A) Detection and quantification of *S*. *mitis* and *P*. *melaninogenica* genomic DNA using pan-*Streptococcus* and pan-*Prevotella* primers. Number of genome copies added to a background of AGS cell genomic DNA is indicated above horizontal bar at the top of each panel. Number of copies/ μl detected in each sample is indicated above positive events (green or blue droplets). Gray, dashed line represents the threshold above which an event was counted as positive. Negative events are depicted in gray. All experiments were performed in duplicate with a representative plot shown. (B) Quantification of *Streptococcus* and *Prevotella* species in biopsy samples from squamous esophagus (ES), Barrett’s esophagus (EB), stomach corpus (SC) and antrum (SA) in individual P12. Number of copies/ μl detected in each sample is indicated above positive events (green or blue droplets). Gray, dashed line represents the threshold above which an event was counted as positive. Negative events are depicted in gray. All experiments were performed in duplicate with a representative plot shown. (C) Quantification of *Streptococcus* and *Prevotella* species in brush samples from ES, EB, SC and SA in individual P12.
(TIF)

S4 Fig Correlation of *Streptococcus*:*Prevotella* ratio with participant demographics and Barrett’s esophagus risk factors. (A) Relationship of hiatal hernia length and waist to hip ratio. Strength of association between these two variables was determined by Pearson’s correlation test with correlation coefficient squared (r^2^) value and p value as indicated. (B) Relationship of *Streptococcus* to *Prevotella* ratio at each anatomic site and participant age. Association between variables was determined by Pearson’s correlation test with r^2^ and p values as indicated. (C) Relationship of *Streptococcus* to *Prevotella* ratio at each anatomic site and Barrett’s segment length. Association between variables was determined by Pearson’s correlation test with r^2^ and p values as indicated. (D) *Streptococcus* to *Prevotella* ratio at each anatomic site and clinical diagnosis determined by histological assessment of Barrett’s esophagus biopsy samples. LG = low grade HG = high grade. (E) *Streptococcus* to *Prevotella* ratio at each anatomic site and participant smoking history.
(TIF)

S1 Table Absolute number of reads per OTU detected in brush and biopsy samples. (DOCX)

S2 Table Absolute number of OTU reads detected in technical replicate biopsy samples. (DOCX)

S3 Table Change in OTU relative abundance over time. (DOCX)
