# Supplementary figures and images for: Bacterial Composition of the Human Upper Gastrointestinal Tract Microbiome Is Dynamic and Associated with Genomic Instability in a Barrett’s Esophagus Cohort
Source: PLoS One. 2015 Jun 15;10(6):e0129055. doi: 10.1371/journal.pone.0129055 (PMC4468150; doi:10.1371/journal.pone.0129055)

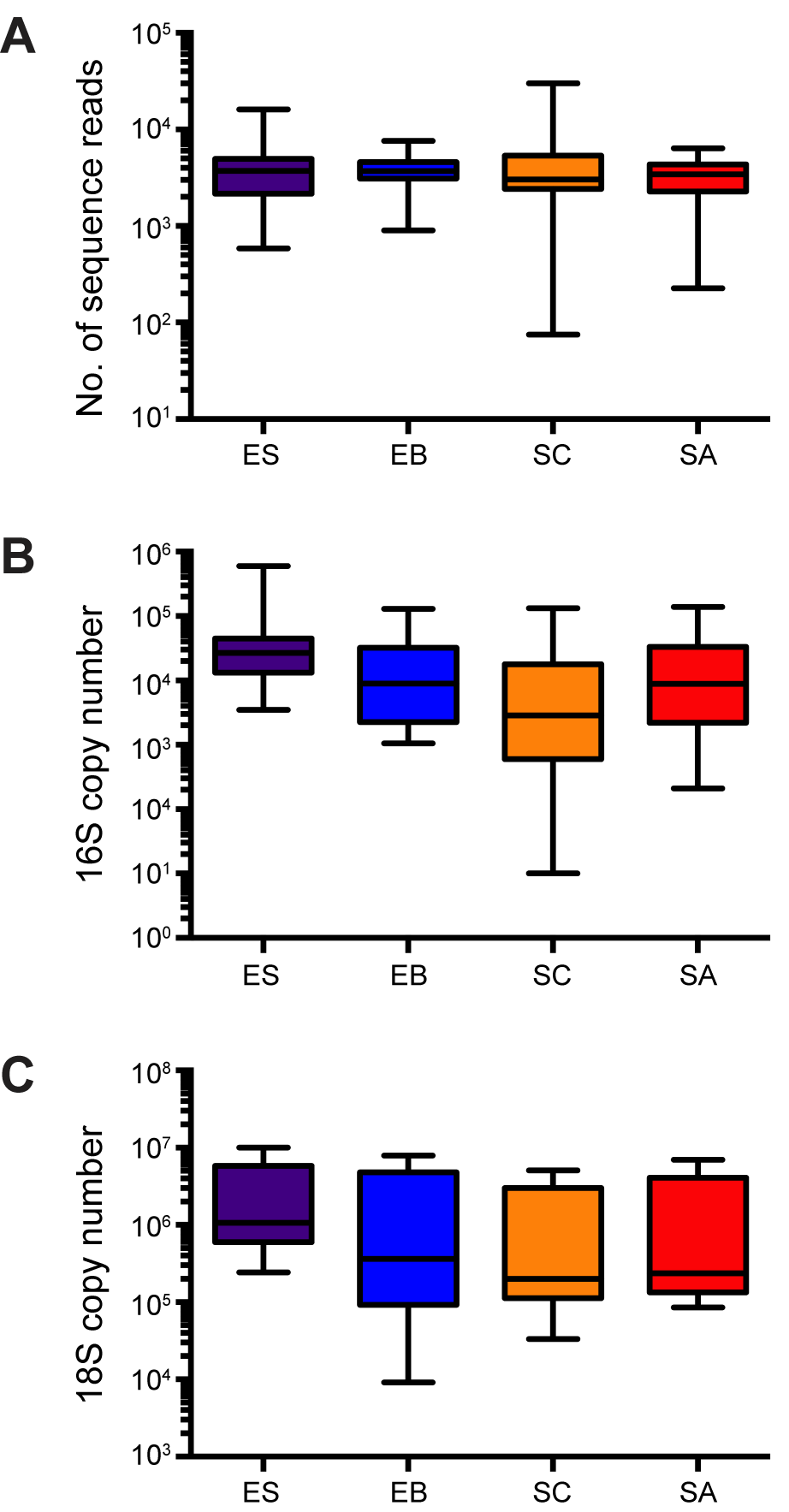

Supplement: S1 Fig — (A) Number of read counts from all study participants per site sampled. Statistical difference between sites was measured by Friedman rank sum test (p = 0.55). (B) Copy number of bacterial 16S rRNA per site sampled, as measured by qPCR. Statistical difference between sites was measured by Friedman rank sum test (p = 0.004). (C) Copy number of human 18S rRNA per site sampled, as measured by qPCR. Statistical difference between sites was measured by Friedman rank sum test (p = 0.001) (TIF) [file pone.0129055.s001.tif]

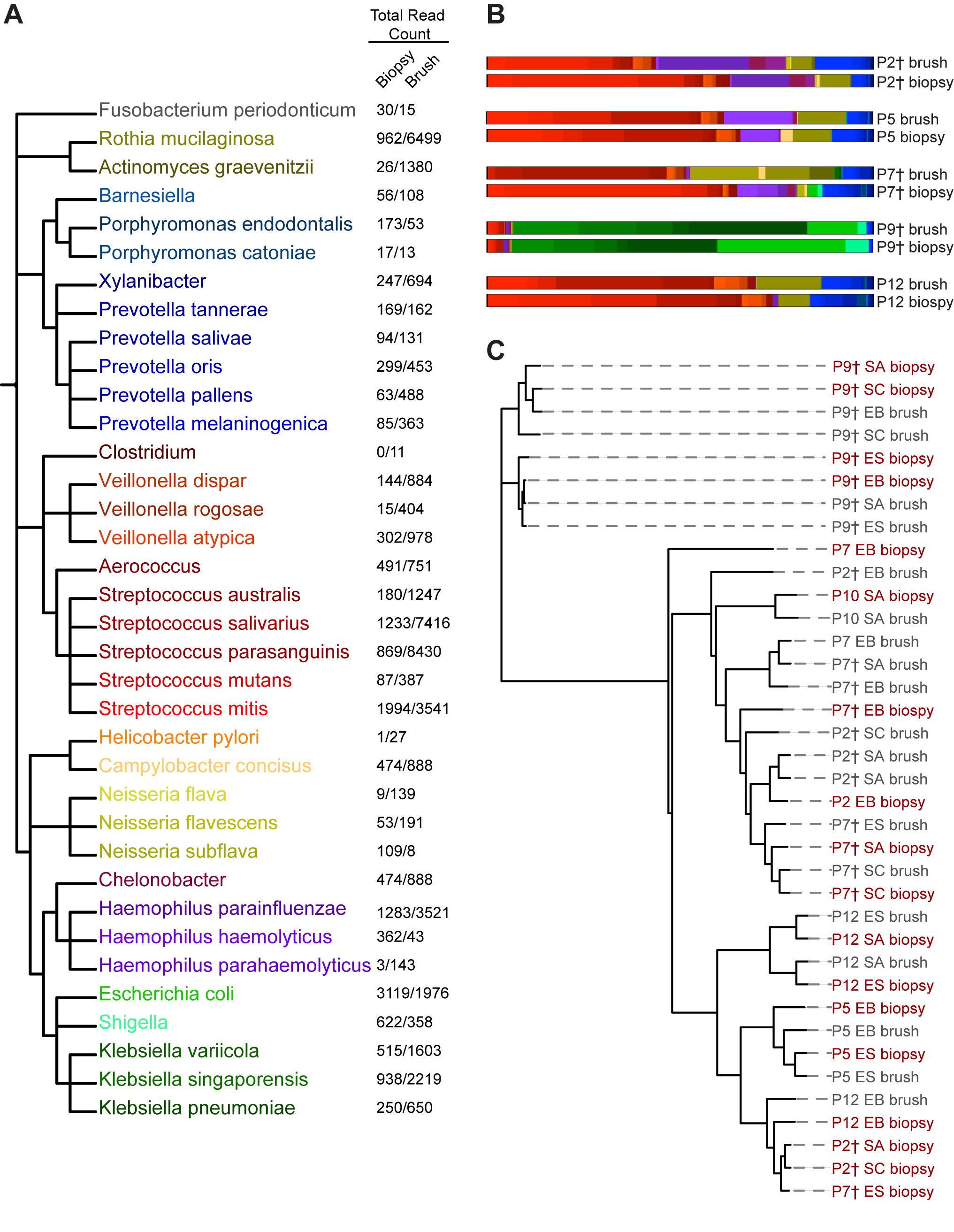

Supplement: S2 Fig — (A) Phylogenetic relationship of the top 45 OTUs detected in samples where both a brush and biopsy specimen was available. Numbers in column represent the total number of reads detected for a given species or genera in brush and biopsy samples. (B) Combined species/genera-level profiles of top 45 OTUs detected by 454 sequencing at all four sites sampled via upper endoscopy in indicated participants. Data are color-coded according to scheme presented in (A). Species reads were normalized to the total number of reads per corresponding site in a given individual.† Denotes samples collected at a second time point (P2 [t = 4 months]; P7 [t = 2 years]; P9 [t = 3 years]). (C) Cluster analysis of KR distances between microbial communities detected in brush or biopsy samples of indicated individuals. (TIF) [file pone.0129055.s002.tif]

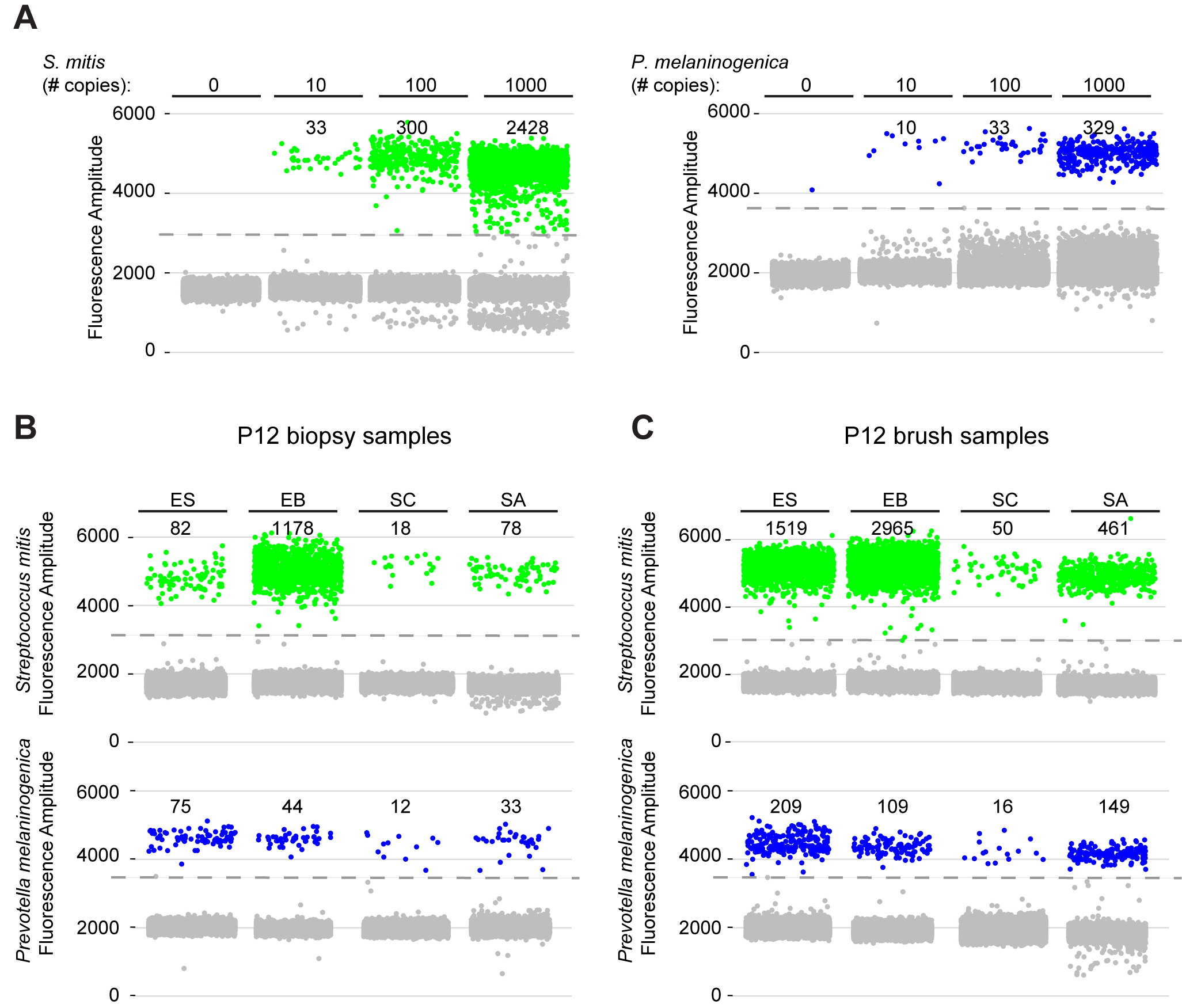

Supplement: S3 Fig — (A) Detection and quantification of S. mitis and P. melaninogenica genomic DNA using pan-Streptococcus and pan-Prevotella primers. Number of genome copies added to a background of AGS cell genomic DNA is indicated above horizontal bar at the top of each panel. Number of copies/ μl detected in each sample is indicated above positive events (green or blue droplets). Gray, dashed line represents the threshold above which an event was counted as positive. Negative events are depicted in gray. All experiments were performed in duplicate with a representative plot shown. (B) Quantification of Streptococcus and Prevotella species in biopsy samples from squamous esophagus (ES), Barrett’s esophagus (EB), stomach corpus (SC) and antrum (SA) in individual P12. Number of copies/ μl detected in each sample is indicated above positive events (green or blue droplets). Gray, dashed line represents the threshold above which an event was counted as positive. Negative events are depicted in gray. All experiments were performed in duplicate with a representative plot shown. (C) Quantification of Streptococcus and Prevotella species in brush samples from ES, EB, SC and SA in individual P12. (TIF) [file pone.0129055.s003.tif]

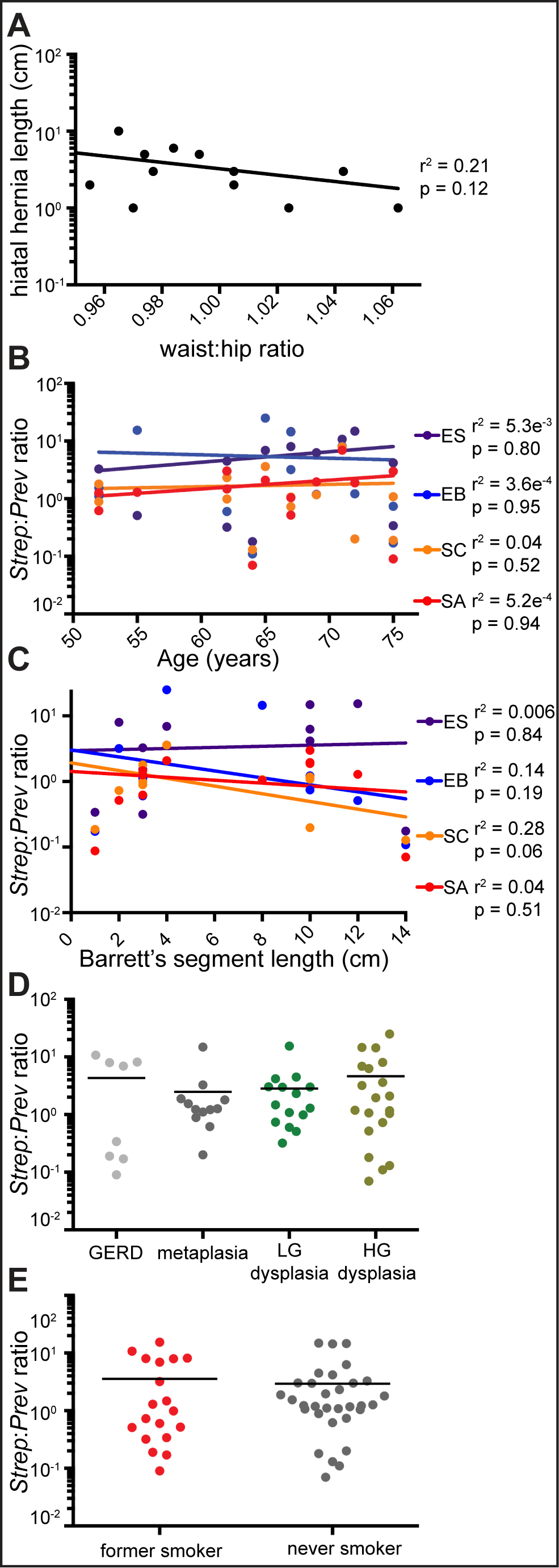

Supplement: S4 Fig — (A) Relationship of hiatal hernia length and waist to hip ratio. Strength of association between these two variables was determined by Pearson’s correlation test with correlation coefficient squared (r2) value and p value as indicated. (B) Relationship of Streptococcus to Prevotella ratio at each anatomic site and participant age. Association between variables was determined by Pearson’s correlation test with r2 and p values as indicated. (C) Relationship of Streptococcus to Prevotella ratio at each anatomic site and Barrett’s segment length. Association between variables was determined by Pearson’s correlation test with r2 and p values as indicated. (D) Streptococcus to Prevotella ratio at each anatomic site and clinical diagnosis determined by histological assessment of Barrett’s esophagus biopsy samples. LG = low grade HG = high grade. (E) Streptococcus to Prevotella ratio at each anatomic site and participant smoking history. (TIF) [file pone.0129055.s004.tif]
