# Supplementary material for: Bacterial Composition of the Human Upper Gastrointestinal Tract Microbiome Is Dynamic and Associated with Genomic Instability in a Barrett’s Esophagus Cohort
Source: PLoS One. 2015 Jun 15;10(6):e0129055. doi: 10.1371/journal.pone.0129055 (PMC4468150; doi:10.1371/journal.pone.0129055)
Supplement: S1 Table — (DOCX) [file pone.0129055.s005.docx]

**S1 Table. Absolute number of reads per OTU detected in brush and biopsy samples**

| **Species** | **P2^†^ biopsy** | **P2^†^ brush** | **P5 biopsy** | **P5 brush** | **P7^†^ biopsy** | **P7^†^ brush** | **P9^†^ biopsy** | **P9^†^ brush** | **P12 biopsy** | **P12 brush** |
| --- | --- | --- | --- | --- | --- | --- | --- | --- | --- | --- |
| *Streptococcus mitis/oralis* | 2144 | 3080 | 37 | 879 | 3197 | 0 | 77 | 174 | 747 | 291 |
| *S. parasanguinis* | 78 | 367 | 15 | 285 | 33 | 6488 | 40 | 54 | 203 | 1236 |
| *Streptococcus salivarius* | 185 | 283 | 131 | 2802 | 209 | 1376 | 92 | 142 | 616 | 2813 |
| *Rothia mucilaginosa* | 515 | 778 | 63 | 1698 | 21 | 2263 | 34 | 50 | 329 | 1710 |
| *K. pneumoniae/variicola* | 0 | 0 | 0 | 0 | 0 | 125 | 2054 | 4521 | 0 | 0 |
| *Streptococcus mitis* | 779 | 935 | 31 | 2051 | 462 | 3 | 51 | 91 | 671 | 461 |
| *Escherichia coli* | 0 | 0 | 0 | 15 | 171 | 26 | 2948 | 1934 | 0 | 1 |
| *H. parainfluenzae* | 979 | 3393 | 1 | 0 | 165 | 1 | 92 | 117 | 46 | 10 |
| *Streptococcus* | 429 | 697 | 90 | 1522 | 304 | 71 | 70 | 178 | 334 | 1050 |
| *Prevotellaceae* | 190 | 1403 | 39 | 407 | 278 | 570 | 64 | 113 | 96 | 470 |
| *Klebsiella* | 0 | 0 | 0 | 0 | 0 | 40 | 957 | 2511 | 0 | 0 |
| *Neisseria flava/sicca* | 2 | 4 | 0 | 0 | 1 | 3444 | 0 | 0 | 0 | 0 |
| *Klebsiella singaporensis* | 0 | 0 | 0 | 0 | 0 | 0 | 938 | 2219 | 0 | 0 |
| *Haemophilus* | 27 | 101 | 62 | 2419 | 364 | 10 | 1 | 2 | 1 | 1 |
| *Klebsiella variicola* | 0 | 0 | 0 | 0 | 0 | 217 | 515 | 1386 | 0 | 0 |
| *Actinomycetales* | 1 | 0 | 0 | 0 | 1 | 2023 | 2 | 2 | 0 | 2 |
| *Paucimonas* | 1 | 2 | 0 | 0 | 35 | 1900 | 0 | 1 | 22 | 19 |
| *Phytobacter* | 1 | 0 | 0 | 4 | 44 | 1071 | 116 | 255 | 15 | 3 |
| *Streptococcus australis* | 64 | 109 | 8 | 125 | 36 | 896 | 11 | 9 | 61 | 108 |
| *A. graevenitzii* | 23 | 115 | 1 | 7 | 0 | 1254 | 2 | 4 | 0 | 0 |
| *Chelonobacter* | 278 | 612 | 2 | 163 | 143 | 17 | 43 | 89 | 8 | 7 |
| *Veillonella atypica* | 110 | 246 | 19 | 234 | 27 | 113 | 4 | 6 | 142 | 379 |
| *Aerococcus* | 244 | 162 | 8 | 219 | 149 | 138 | 21 | 31 | 69 | 201 |
| *Prevotella* | 44 | 304 | 5 | 105 | 114 | 150 | 15 | 76 | 57 | 301 |
| *Veillonella dispar* | 62 | 334 | 7 | 269 | 26 | 53 | 7 | 24 | 42 | 204 |
| *Pasteurellaceae* | 155 | 766 | 0 | 0 | 26 | 30 | 19 | 7 | 0 | 0 |
| *Shigella* | 0 | 0 | 1 | 15 | 89 | 1 | 532 | 342 | 0 | 0 |
| *Veillonella* | 38 | 118 | 6 | 83 | 54 | 309 | 1 | 7 | 65 | 293 |
| *Xylanibacter* | 71 | 164 | 9 | 115 | 118 | 185 | 19 | 54 | 57 | 176 |
| *V. atypica/dispar* | 5 | 21 | 0 | 12 | 2 | 739 | 0 | 0 | 24 | 131 |
| *Klebsiella pneumoniae* | 0 | 0 | 0 | 0 | 0 | 0 | 250 | 650 | 0 | 0 |
| *N. flavescens/subflava* | 35 | 573 | 0 | 0 | 11 | 212 | 16 | 12 | 0 | 0 |
| *G. paraadiacens* | 0 | 2 | 0 | 2 | 1 | 759 | 0 | 1 | 2 | 2 |
| *Prevotella oris* | 0 | 1 | 7 | 163 | 109 | 11 | 0 | 0 | 183 | 278 |
| *Fusobacterium* | 0 | 0 | 1 | 11 | 13 | 684 | 0 | 1 | 11 | 11 |
| *Leptotrichia buccalis* | 1 | 9 | 0 | 0 | 18 | 666 | 0 | 1 | 1 | 1 |
| *E. asburiae/hormaechei* | 0 | 0 | 0 | 0 | 1 | 5 | 255 | 351 | 0 | 0 |
| *Prevotella pallens* | 3 | 20 | 0 | 0 | 59 | 468 | 1 | 0 | 0 | 0 |
| *Campylobacter concisus* | 53 | 43 | 20 | 33 | 43 | 343 | 0 | 2 | 0 | 4 |
| *Streptococcus oralis* | 18 | 21 | 5 | 274 | 31 | 24 | 0 | 6 | 31 | 92 |
| *E.hormaechei/K. variicola* | 0 | 0 | 0 | 0 | 0 | 484 | 0 | 0 | 0 | 0 |
| *Streptococcus mutans* | 2 | 0 | 69 | 26 | 8 | 351 | 0 | 0 | 8 | 10 |
| *Moryella indoligenes* | 13 | 52 | 0 | 0 | 1 | 392 | 4 | 1 | 2 | 0 |
| *P. melaninogenica* | 23 | 184 | 1 | 4 | 53 | 152 | 4 | 21 | 4 | 2 |
| *Firmicutes* | 71 | 84 | 5 | 49 | 137 | 3 | 4 | 13 | 10 | 50 |

Only the top 45 OTUs are represented

**^†^** Denotes samples collected at a second time point (P2 [t=4 months]; P7 [t=2 years]; P9 [t=3 years])
