# Supplementary material for: Bacterial Composition of the Human Upper Gastrointestinal Tract Microbiome Is Dynamic and Associated with Genomic Instability in a Barrett’s Esophagus Cohort
Source: PLoS One. 2015 Jun 15;10(6):e0129055. doi: 10.1371/journal.pone.0129055 (PMC4468150; doi:10.1371/journal.pone.0129055)
Supplement: S2 Table — (DOCX) [file pone.0129055.s006.docx]

**S2 Table. Absolute number of OTU reads detected in technical replicate biopsy samples**

| **Species** | **P2 EB**  **rep#1** | **P2 EB**  **rep#2** | **P6 SA**  **rep#1** | **P6 SA**  **rep#2** | **P6 SC**  **rep#1** | **P6 SC**  **rep#2** | **P6 EB**  **rep#1** | **P6 EB**  **rep#2** |
| --- | --- | --- | --- | --- | --- | --- | --- | --- |
| *Helicobacter pylori* | 0 | 0 | 841 | 735 | 6000 | 3002 | 208 | 0 |
| *S. mitis/oralis* | 1223 | 1451 | 79 | 24 | 2 | 0 | 63 | 0 |
| *Escherichia coli* | 0 | 0 | 657 | 1121 | 17 | 6 | 341 | 1 |
| *H. parainfluenzae* | 374 | 292 | 182 | 109 | 7 | 0 | 177 | 0 |
| *Streptococcus mitis* | 416 | 439 | 29 | 12 | 2 | 0 | 23 | 0 |
| *Rothia mucilaginosa* | 320 | 427 | 22 | 14 | 2 | 0 | 29 | 1 |
| *Neisseria subflava* | 2 | 0 | 145 | 46 | 0 | 0 | 561 | 0 |
| *Clostridium* | 0 | 0 | 360 | 178 | 12 | 2 | 158 | 0 |
| *Streptococcus* | 167 | 374 | 56 | 26 | 2 | 0 | 75 | 0 |
| *Veillonella rogosae* | 4 | 0 | 139 | 74 | 9 | 0 | 301 | 0 |
| *Veillonella atypica* | 143 | 47 | 86 | 37 | 2 | 2 | 172 | 0 |
| *Veillonella dispar* | 42 | 15 | 102 | 50 | 2 | 1 | 134 | 0 |
| *Veillonella* | 108 | 46 | 64 | 15 | 4 | 0 | 90 | 0 |
| *P. catoniae* | 0 | 0 | 34 | 11 | 1 | 1 | 273 | 0 |
| *P. melaninogenica* | 7 | 3 | 67 | 36 | 0 | 0 | 179 | 0 |
| *Gemella haemolysans* | 92 | 176 | 1 | 4 | 0 | 0 | 2 | 0 |
| *Chelonobacter* | 49 | 86 | 3 | 55 | 2 | 2 | 62 | 0 |
| *Neisseria flava* | 105 | 144 | 4 | 3 | 0 | 0 | 2 | 0 |
| *Aerococcus* | 55 | 163 | 2 | 5 | 0 | 0 | 30 | 0 |
| *Neisseria sicca* | 186 | 20 | 11 | 1 | 2 | 0 | 4 | 0 |
| *Gemella* | 162 | 8 | 12 | 2 | 0 | 0 | 5 | 0 |
| *C. perfringens* | 0 | 0 | 83 | 39 | 1 | 1 | 63 | 0 |
| *Shigella* | 0 | 0 | 51 | 91 | 0 | 0 | 32 | 0 |
| *S. parasanguinis* | 99 | 66 | 0 | 0 | 0 | 0 | 8 | 0 |
| *A. odontolyticus* | 4 | 0 | 38 | 30 | 2 | 0 | 98 | 1 |
| *S. australis* | 111 | 10 | 4 | 3 | 3 | 0 | 8 | 0 |
| *S. salivarius* | 25 | 25 | 18 | 5 | 2 | 1 | 59 | 0 |
| *Prevotellaceae* | 60 | 50 | 5 | 3 | 0 | 0 | 8 | 0 |
| *Neisseria flavescens* | 1 | 2 | 22 | 7 | 0 | 0 | 87 | 1 |
| *Sarcina* | 0 | 0 | 3 | 54 | 5 | 2 | 49 | 1 |
| *Gemella sanguinis* | 41 | 16 | 21 | 4 | 1 | 0 | 25 | 0 |
| *Neisseria flava/sicca* | 58 | 44 | 1 | 1 | 0 | 0 | 1 | 0 |
| *Firmicutes* | 43 | 10 | 11 | 6 | 0 | 0 | 30 | 0 |
| *Prevotella* | 30 | 24 | 6 | 8 | 0 | 0 | 30 | 0 |
| *Oribacterium sinus* | 0 | 1 | 14 | 8 | 6 | 0 | 61 | 0 |
| *Phytobacter* | 0 | 0 | 4 | 31 | 8 | 6 | 40 | 0 |
| *G.adiacens* | 35 | 12 | 20 | 0 | 1 | 0 | 21 | 0 |
| *Catonella morbi* | 0 | 0 | 15 | 4 | 0 | 0 | 68 | 0 |
| *Leptotrichia buccalis* | 2 | 0 | 7 | 9 | 1 | 0 | 64 | 0 |
| *Xylanibacter* | 7 | 20 | 0 | 8 | 1 | 0 | 45 | 0 |
| *N. flavescens/subflava* | 0 | 0 | 3 | 2 | 0 | 0 | 73 | 0 |
| *F. periodonticum* | 0 | 0 | 14 | 5 | 0 | 0 | 57 | 0 |
| *Barnesiella* | 1 | 1 | 2 | 4 | 0 | 0 | 54 | 0 |
| *Streptococcus oralis* | 50 | 4 | 3 | 0 | 0 | 0 | 5 | 0 |
| *C. concisus* | 14 | 7 | 4 | 6 | 0 | 0 | 27 | 0 |

Only the top 45 OTUs are represented
