# Supplementary material for: Bacterial Composition of the Human Upper Gastrointestinal Tract Microbiome Is Dynamic and Associated with Genomic Instability in a Barrett’s Esophagus Cohort
Source: PLoS One. 2015 Jun 15;10(6):e0129055. doi: 10.1371/journal.pone.0129055 (PMC4468150; doi:10.1371/journal.pone.0129055)
Supplement: S3 Table — (DOCX) [file pone.0129055.s007.docx]

**S3 Table. Change in OTU relative abundance over time**

| **Site** | **Species** | **Participant** | **Relative abundance at 1^st^ time point (t_1_)*** | **Relative abundance at 2^nd^ time point (t_2_)*** | **%Δ (t_2_-t_1_)** |
| --- | --- | --- | --- | --- | --- |
| S**tomach antrum** | *Streptococcus salivarius* | P9 | 38.9 | 0.9 | -38.0 |
|  | *Streptococcus* | P9 | 15 | 1.6 | -13.4 |
|  | *Streptococcus parasanguinis* | P9 | 7.1 | 0.8 | -6.2 |
|  | *Klebsiella singaporensis* | P9 | 2.2 | 11 | +8.8 |
|  | *Klebsiella* | P9 | 1.3 | 10.8 | +9.5 |
|  | *Escherichia coli* | P9 | 2.7 | 13.7 | +11.0 |
|  | *Klebsiella pneumoniae/variicola* | P9 | 3.5 | 18.5 | +14.9 |
|  | *Rothia mucilaginosa* | P2 | 14.1 | 2.7 | -11.5 |
|  | *Prevotellaceae* | P2 | 9.6 | 1.7 | -7.9 |
|  | *Veillonella dispar* | P2 | 7.1 | 0.3 | -6.8 |
|  | *Prevotella melaninogenica* | P2 | 5.5 | 0.1 | -5.4 |
|  | *Haemophilus parainfluenzae* | P2 | 0.8 | 6.3 | +5.5 |
|  | *Streptococcus mitis* | P2 | 3.4 | 16.9 | +13.5 |
|  | *Streptococcus mitis/oralis* | P2 | 6.8 | 49.6 | +42.8 |
|  | *Haemophilus* | P7 | 4.3 | 9.3 | +5.0 |
| **Stomach corpus** | *Actinomyces odontolyticus* | P9 | 7.1 | 0.1 | -7.0 |
|  | *Bifidobacterium* | P9 | 7.8 | 0.1 | -7.7 |
|  | *Streptococcus* | P9 | 7.8 | 0.6 | -7.2 |
|  | *Escherichia coli* | P9 | 0 | 5.6 | +5.6 |
|  | *Klebsiella variicola* | P9 | 0 | 7 | +7.0 |
|  | *Klebsiella* | P9 | 3.5 | 14.5 | +10.9 |
|  | *Klebsiella pneumoniae/variicola* | P9 | 1.4 | 28.3 | +26.9 |
|  | *Lactobacillus reuteri* | P2 | 23.8 | 0 | -23.8 |
|  | *Paralactobacillus* | P2 | 13.9 | 0 | -13.9 |
|  | *Streptococcus mitis* | P2 | 2.3 | 23 | +20.7 |
|  | *Streptococcus mitis/oralis* | P2 | 5 | 50.2 | +45.1 |
|  | *Rothia mucilaginosa* | P7 | 20.3 | 1 | -19.3 |
|  | *Prevotellaceae* | P7 | 3.6 | 9.7 | +6.1 |
|  | *Streptococcus mitis/oralis* | P7 | 6.4 | 19.7 | +13.4 |
| **Barrett’s esophagus** | *Streptococcus salivarius* | P9 | 38 | 0.4 | -37.6 |
|  | *Streptococcus* | P9 | 10.9 | 0.3 | -10.6 |
|  | *Streptococcus parasanguinis* | P9 | 5.7 | 0.2 | -5.6 |
|  | *Klebsiella variicola* | P9 | 0.1 | 8.4 | +8.3 |
|  | *Escherichia coli* | P9 | 0.1 | 10.7 | +10.6 |
|  | *Klebsiella* | P9 | 1.1 | 14.2 | +13.1 |
|  | *Klebsiella pneumoniae/variicola* | P9 | 7.4 | 37.1 | +29.7 |
|  | *Neisseria flavescens/subflava* | P2 | 0 | 5.5 | +5.5 |
|  | *Pasteurellaceae* | P2 | 0.5 | 5.9 | +5.4 |
|  | *Prevotellaceae* | P2 | 1.4 | 9.1 | +7.7 |
|  | *Prevotella* | P7 | 10.1 | 3.8 | -6.2 |
|  | *Streptococcus mitis/oralis* | P7 | 8.6 | 13.7 | +5.1 |
|  | *Haemophilus* | P7 | 5.4 | 13.5 | +8.1 |
| **Squamous esophagus** | *Streptococcus salivarius* | P9 | 43.8 | 1.4 | -42.3 |
|  | *Streptococcus* | P9 | 12.3 | 0.5 | -11.8 |
|  | *Streptococcus mitis/oralis* | P9 | 8.7 | 0.1 | -8.6 |
|  | *Streptococcus parasanguinis* | P9 | 7.7 | 0.2 | -7.5 |
|  | *Shigella* | P9 | 0 | 9.6 | +9.6 |
|  | *Escherichia coli* | P9 | 0.2 | 60 | 59.8 |
|  | *Rothia mucilaginosa* | P2 | 29.2 | 8.9 | -20.3 |
|  | *Streptococcus parasanguinis* | P2 | 12.6 | 1.3 | -11.3 |
|  | *Streptococcus salivarius* | P2 | 12.1 | 3 | -9.1 |
|  | *Chelonobacter* | P2 | 0.2 | 4.9 | +4.8 |
|  | *Haemophilus parainfluenzae* | P2 | 1.9 | 15.4 | +13.6 |
|  | *Streptococcus mitis/oralis* | P2 | 8.6 | 23.7 | +15.1 |
|  | *Prevotellaceae* | P7 | 10.1 | 3.4 | -6.6 |
|  | *Haemophilus* | P7 | 4.9 | 9.9 | +5.0 |
|  | *Streptococcus mitis/oralis* | P7 | 7.6 | 25.9 | +18.3 |

*Relative abundance at the first and second time point was calculated by dividing the species specific read counts by the total number of counts detected at respective sites within individuals and multiplied by 100%. Only OTUs that exhibited an increase of decrease of $\geq$ 5% between the two time points sampled are represented.
